# Integrated multiomics reveals starvation-driven keratin degradation and persistence in *Fervidobacterium islandicum* AW-1

**DOI:** 10.1016/j.isci.2026.115755

**Published:** 2026-04-20

**Authors:** Jae-Yoon Sung, Ji-Yeon Kim, Hyeon-Su Jin, Je-Hyun Baek, Nicole Enjeh Kim, Hyun Ho Song, Seong-Hun Bong, Yong-Jik Lee, Byoung-Chan Kim, Do Yup Lee, Dong-Woo Lee

**Affiliations:** 1 Department of Biotechnology, Yonsei University, Seoul 03722, South Korea; 2 Department of Environment and Energy Engineering, Gwangju Institute of Science and Technology (GIST), Gwangju 61005, South Korea; 3 R&D Center for Clinical Mass Spectrometry, Seegene Medical Foundation, Seoul 04805, South Korea; 4 Department of Agricultural Biotechnology, Center for Food and Bioconvergence, Research Institute for Agricultural and Life Sciences, Seoul National University, Seoul 08826, South Korea; 5 Department of Bio-Cosmetics, Seowon University, Cheongju 28674, South Korea; 6 HealthBiome, Inc., 48, Yuseong-daero 1184beon-gil, Yuseong-gu, Daejeon 34109, South Korea

**Keywords:** biological sciences

## Abstract

Keratin is one of the most recalcitrant biopolymers due to its dense disulfide crosslinks; yet, how microbial keratin degradation is regulated under nutrient limitation remains poorly understood. Here, we integrated time-resolved transcriptomics, proteomics, and metabolomics to investigate starvation-driven keratin utilization in the extremophilic bacterium *Fervidobacterium islandicum* AW-1. Although keratinolytic proteases are constitutively expressed, starvation induces a coordinated regulatory program that couples keratin degradation to stress adaptation. Under nutrient limitation, cells degraded keratin through localized membrane-associated proteases, while redox-mediated sulfitolysis and Fe-S cluster biogenesis facilitated disulfide bond cleavage and redox balance. Metabolic rewiring favored the Entner-Doudoroff pathway and the reverse TCA cycle, conserving energy under oligotrophic conditions. Starvation further activated the stringent response and cyclic-di-GMP-associated signaling, promoting biofilm formation, persistence-like behavior, and substrate colonization. Together, these findings propose a systems-level model linking keratin degradation to regulatory and metabolic networks that support microbial persistence in extreme environments and keratin waste valorization.

## Highlights

• Time-resolved multi-omics reveal starvation-driven keratin degradation networks
• Membrane-associated proteases and sulfitolysis enable localized keratin processing
• Stringent response and c-di-GMP circuits coordinate metabolic shift and persistence
• Integrative model links proteolysis, redox metabolism, and survival strategy

## Graphical abstract

**Figure:**
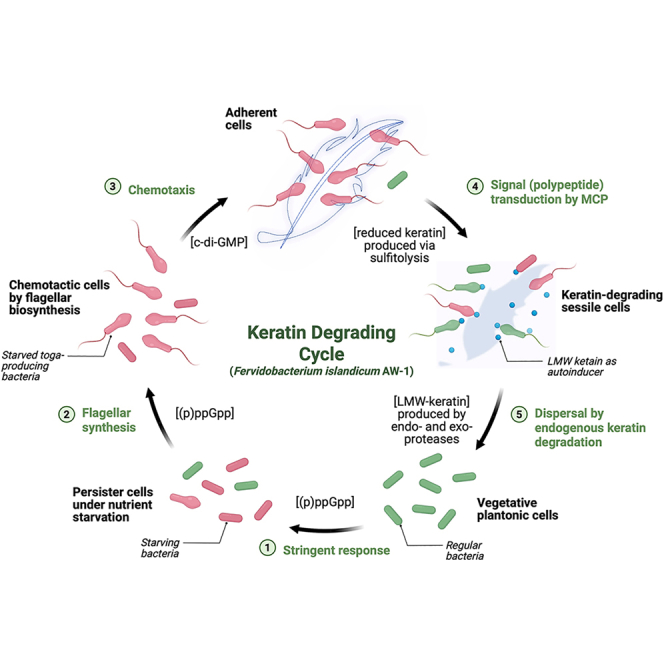

## Introduction

Microbes have evolved diverse strategies to endure nutrient limitation, including sporulation, biofilm formation, persister cell formation, and metabolic plasticity.^1^^,^^2^^,^^3^^,^^4^ These adaptations enable survival in extreme environments and have contributed to microbial ecological success over billions of years.^5^^,^^6^ Gram-positive Bacillota (formerly Firmicutes) often form dormant spores under starvation,^7^ whereas Gram-negative Pseudomonadota (Proteobacteria) activate chemotaxis, motility, and biofilm formation to enhance nutrient acquisition and stress tolerance.^8^^,^^9^ Another critical adaptation is the ability to utilize recalcitrant biopolymers such as lignocellulose, chitin, and keratin as alternative nutrient sources.^10^

Keratin, a fibrous structural protein in feathers, hair, and skin, is among the most degradation-resistant biopolymers due to its dense hydrogen bonding and extensive disulfide cross-linking.^11^ Nevertheless, specialized bacteria and fungi have evolved enzymatic systems capable of keratin breakdown, enabling survival in oligotrophic environments.^12^^,^^13^^,^^14^ Recent multi-omics studies of feather degradation have cataloged transcriptional and proteomic changes associated with keratinolytic and sulfitolytic enzymes.^15^^,^^16^^,^^17^^,^^18^ However, these studies have largely focused on individual omics layers or enzyme inventories, rather than elucidating the upstream physiological triggers and regulatory programs that initiate and coordinate keratin utilization under nutrient stress.

Previously, we identified the extremophilic anaerobe *Fervidobacterium islandicum* AW-1 as capable of degrading native keratin at 70°C.^19^^,^^20^ Unlike canonical extracellular proteolysis, keratinolysis by *F*. *islandicum* AW-1 involves membrane-associated proteases and redox-mediated sulfitolysis, a process that disrupts keratin disulfide bonds to facilitate peptide backbone hydrolysis.^21^ The starvation-associated upregulation of membrane-bound metalloproteases and sulfur metabolism genes further suggests that keratin degradation is embedded within a broader stress-adaptive program rather than functioning as an isolated catabolic response.^12^^,^^22^^,^^23^^,^^24^

Despite these insights, the molecular signals, metabolic reorganization, and regulatory circuits that coordinate keratin utilization during starvation remain poorly understood. Here, we integrate time-resolved transcriptomic, proteomic, and metabolomic analyses to show that keratin degradation in *F*. *islandicum* AW-1 represents a core starvation survival strategy rather than a substrate-specific response. This program couples membrane-associated proteolysis and sulfur assimilation with stress-responsive signaling and a dynamic transition between motility-driven dispersal and biofilm-associated persistence, mediated by cyclic-di-GMP signaling and stringent response pathways. Together, our findings provide a mechanistic framework for microbial persistence in keratin-rich, soluble nutrient-poor environments and highlight new opportunities for keratin waste valorization.

## Results

### Membrane-associated proteolysis and redox-mediated sulfitolysis enable efficient keratin degradation under starvation

To determine whether keratin serves as a viable nutrient source under starvation, we monitored the growth of *F*. *islandicum* AW-1 and amino acid release during anaerobic incubation with native feathers at 70°C. Feather-grown cells sustained prolonged growth, accompanied by progressive feather biomass loss and the accumulation of both keratin-derived amino acids (Ala, Gly, Ser, Val, and Ile) and essential amino acids (Met, Trp, and Lys) (Figures S1A and S1B; Table S1). Organic acid profiles were comparable between feather- and glucose-grown cultures (lactate, 6 mM; acetate, 3 mM), indicating that keratin can fully support both carbon and nitrogen requirements (Figure S1C).

**Figure 1:**
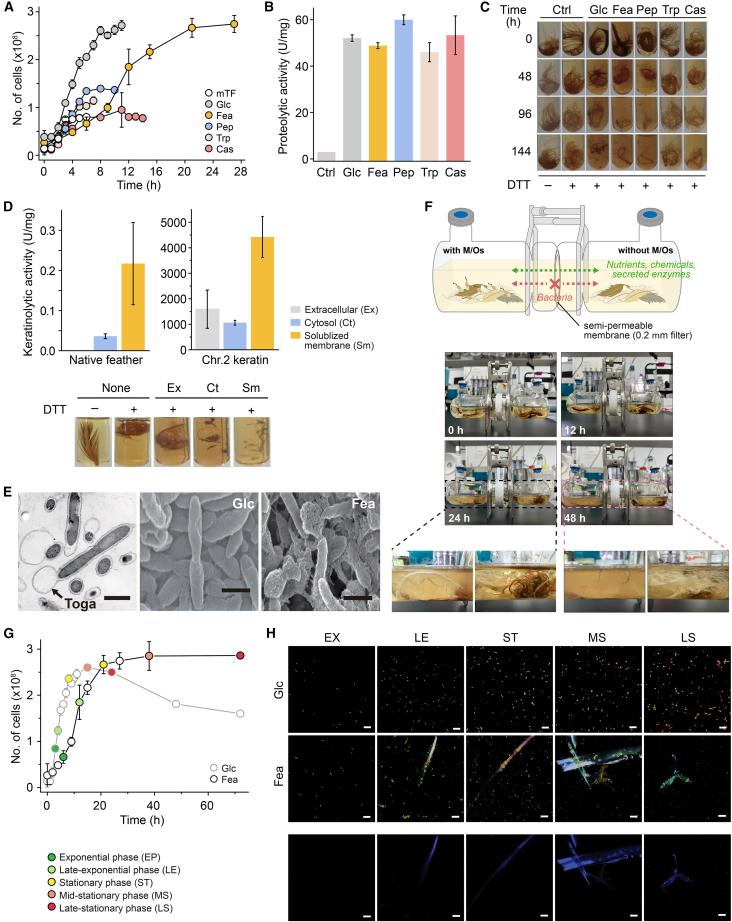
Growth physiology and redox-enhanced keratin degradation by *F*. *islandicum* AW-1 (A) Growth curves in modified TF (mTF) medium supplemented with glucose (Glc), peptone (Pep), tryptone (Trp), casein (Cas), or native feathers (Fea) at 70°C. (B) Total proteolytic activity in crude extracts (0.1 mg/mL) from late-exponential phase cells. (C) Feather degradation by crude extracts with or without 10 mM DTT at 75°C. (D) Keratinolytic activity in extracellular (Ex), cytosolic (Ct), and SM fractions from Fea-grown cells incubated for 24 h on native feathers (left) or recombinant Chr.2 keratin^25^ (right) as substrates. (E) Transmission (left) and SEM (right) images showing smooth rods in Glc-grown cells versus expanded toga envelopes in Fea-grown cells (scale bars, 1 μm). (F) Two-chamber diffusion assay showing that keratin hydrolysis requires direct cell contact. (G) Comparative growth curves in Glc-vs. Fea-supplemented medium. (H) CLSM images showing planktonic Glc-grown cells versus biofilm-like aggregates adhering to feather surfaces in Fea-grown cells. SYTO9 (green) and propidium iodide (PI) indicate live and dead cells, respectively; feather fragments stained with CPM (blue). Scale bars, 10 μm.

Nutrient-dependent comparisons show that glucose, peptone, tryptone, or casein supported rapid early-phase growth, whereas feather-grown cells reached similar or higher final cell densities and accumulated greater levels of free amino acids (Figures 1A and S1D). Total protease activity, measured using casein, was comparable across nutrient conditions, and feather hydrolysis by crude extracts varied minimally under reducing conditions (Figures 1B and 1C). Feather concentration had little effect on degradation efficiency (Figures S1E–S1G), suggesting that keratin does not transcriptionally induce a dedicated protease system. Because keratin’s extensive disulfide bonds impede proteolysis, we tested the effect of dithiothreitol (DTT) on feather solubilization. DTT significantly enhanced amino acid release (Figures 1C, S1D, S1F–S1G), indicating that thiol-mediated sulfitolysis facilitates proteolytic access to keratin’s rigid backbone.

Subcellular fractionation showed negligible keratinolytic activity in extracellular fractions but substantial activity in solubilized membrane (SM) fractions, particularly from feather-grown cells (Figures 1D and S1H). These fractions efficiently degraded both native feathers and recombinant keratins, identifying the SM proteome as the primary site of proteolysis during starvation. A two-chamber diffusion system confirmed that degradation required direct cell-surface contact rather than secreted proteases or diffusible reductants (Figure 1F).

Microscopy revealed distinct starvation-induced morphological changes: scanning electron microscopy (SEM) showed expanded “toga”-like outer envelopes in feather-grown cells, and fatty acid analysis indicated a shift from saturated palmitic acid (C_16:0_; 79% in glucose-grown cells) to unsaturated oleic acid (C_18:1_ *cis*-9, 23%), suggesting increased membrane fluidity (Figure 1E; Table S2). Confocal laser scanning microscopy (CLSM) visualized dense sessile aggregates on feather surfaces in starved cultures, whereas glucose-grown cells remained planktonic and lost viability in stationary phase (Figures 1G and 1H).

Together, these findings indicate that *F*. *islandicum* AW-1 possesses a constitutive proteolytic capacity for keratin degradation, but that efficient keratin utilization is achieved only under starvation through redox-mediated sulfitolysis. Nutrient limitation triggers membrane remodeling, spatial confinement of proteolysis to the cell surface, and biofilm-like aggregation on keratin substrates, optimizing nutrient acquisition in oligotrophic, disulfide-rich environments.

### Feather-specific transcriptome highlights sulfur metabolism, energy optimization, and stress adaptation

**Figure 2:**
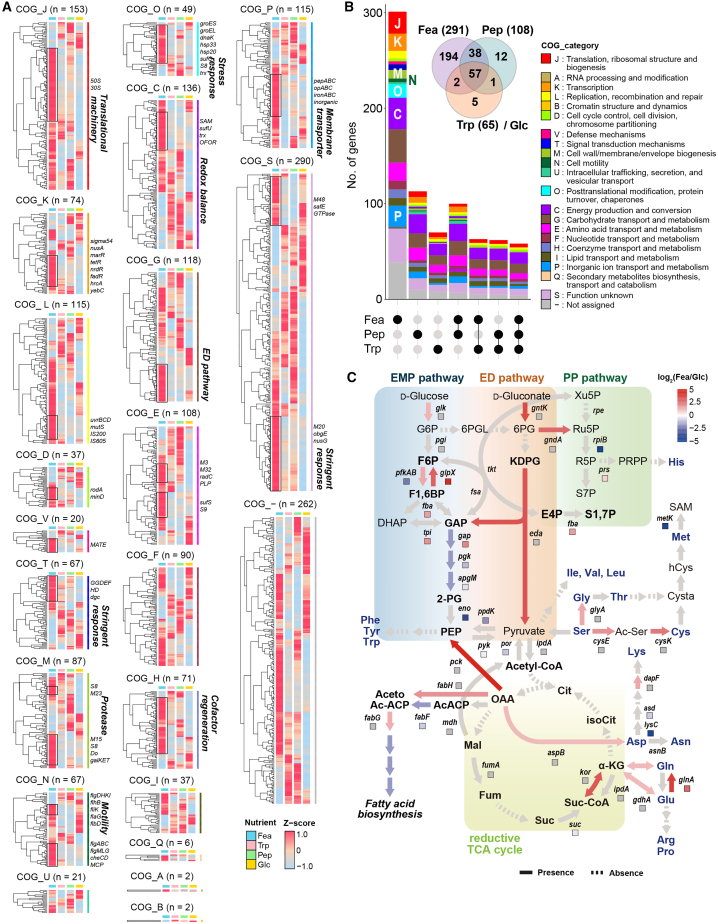
Nutrient-dependent transcriptomic reprogramming highlights sulfur metabolism, membrane-associated proteases, and energy conservation (A) Heatmaps of DEGs in Fea-, Pep-, and Trp-grown cells compared to Glc-grown cells at 8 h, grouped by COG functional classes. Genes involved in sulfur metabolism (*suf*, *trx*, and *isc*), redox regulation, chemotaxis/motility, proteases, and stress responses are annotated. (B) Functional categorization of DEGs by COG categories. The Venn diagram (top) highlights unique and shared DEGs among conditions; the upset plot (bottom) depicts overlaps between conditions. (C) Metabolic pathway mapping showing rewiring from glycolysis (EMP; blue) to the Entner-Doudoroff (ED; red) and reductive TCA pathways (yellow), along with upregulation of sulfur assimilation and organic acid transport. Dotted arrows indicate reactions absent in the *F*. *islandicum* AW-1 genome. Arrows (transcriptomic) and squares (proteomic) are color-coded by relative expression (log_2_FC, Fea vs. Glc, *p* < 0.05).

To identify transcriptional programs supporting keratin utilization, we performed RNA sequencing on *F*. *islandicum* AW-1 during exponential growth (6–8 h) in native feather, glucose, peptone, or tryptone media (Table S3). Among 1,949 annotated coding sequences (GenBank: NZ_CP014334.1),^12^ 336 genes were differentially expressed in feather-grown cells compared to glucose-grown cells, with 291 upregulated and 45 downregulated (fold change ≥ 2, *p* < 0.05) (Figures 2A, 2B, and S2A; Data S1; Sheets 1–3). A subset of differentially expressed genes (DEGs) preferentially associated with feather-dependent growth (*n* = 194) was enriched in four functional categories (Figures 2A, 2B, and S2B; Data S1: Sheet 1): (i) sulfur metabolism and redox homeostasis, including the Suf Fe-S cluster assembly system (*sufB* and *sufC*), cysteine synthase, and radical SAM enzymes (Figures 2, S2A, and S2B); (ii) membrane-associated proteolysis, with induction of metalloproteases (M23 and M48) and cytosolic peptidases (M38, M55, and M16) (Figures S2A and S2C); (iii) nutrient transport systems, such as ABC-type peptide/amino acid transporters and tripartite tricarboxylate transporters; and (iv) stress-responsive regulation, with strong induction of TetR-, MarR-, and FadR-family transcription factors, alongside repression of NADH-sensing regulator Rex.

Metabolic pathway mapping indicated a transcriptional shift away from canonical glycolysis toward the Entner-Doudoroff (ED) pathway, with upregulation of 6-phosphogluconate dehydratase and KDPG aldolase, and downregulation of phosphofructokinase (PFK) (Figures 2C and S2B). Components of the reverse tricarboxylic acid (TCA) cycle, including ATP citrate lyase and ferredoxin-dependent oxidoreductases, were also upregulated, consistent with redistribution of biosynthetic fluxes under energy-limited conditions. Sulfur-linked adaptations are consistent with a role in keratin disulfide bond reduction via redox-mediated sulfitolysis while maintaining intracellular redox balance. Concomitant induction of molecular chaperones (DnaK and GroES), DNA repair proteins, and oxidative stress defenses suggests that keratin degradation imposes proteotoxic and redox stress (Figures 2A, 2B, and S2A). In contrast, ATP-intensive processes such as thiamin biosynthesis (*thiC*, *thiS*, and *thiE*) and heavy metal efflux systems were repressed, suggesting prioritization of energy allocation for survival (Figures 2, S2A, and S2B; Data S1: Sheet 1).

Finally, genes associated with chemotaxis and motility (e.g., *fliC*, *flhA*, methyl-accepting chemotaxis proteins, etc.) were upregulated, consistent with microscopic evidence of enhanced surface colonization during feather utilization (Figures 1F–1H, 2A, and S2A). Collectively, these data outline a starvation-adaptive transcriptional program imposed by keratin utilization that integrates sulfur metabolism, energy-efficient carbon processing, and stress-responsive regulation.

### Coordinated transcriptomic-proteomic shift accompanies the transition from motile growth to sessile persistence

**Figure 3:**
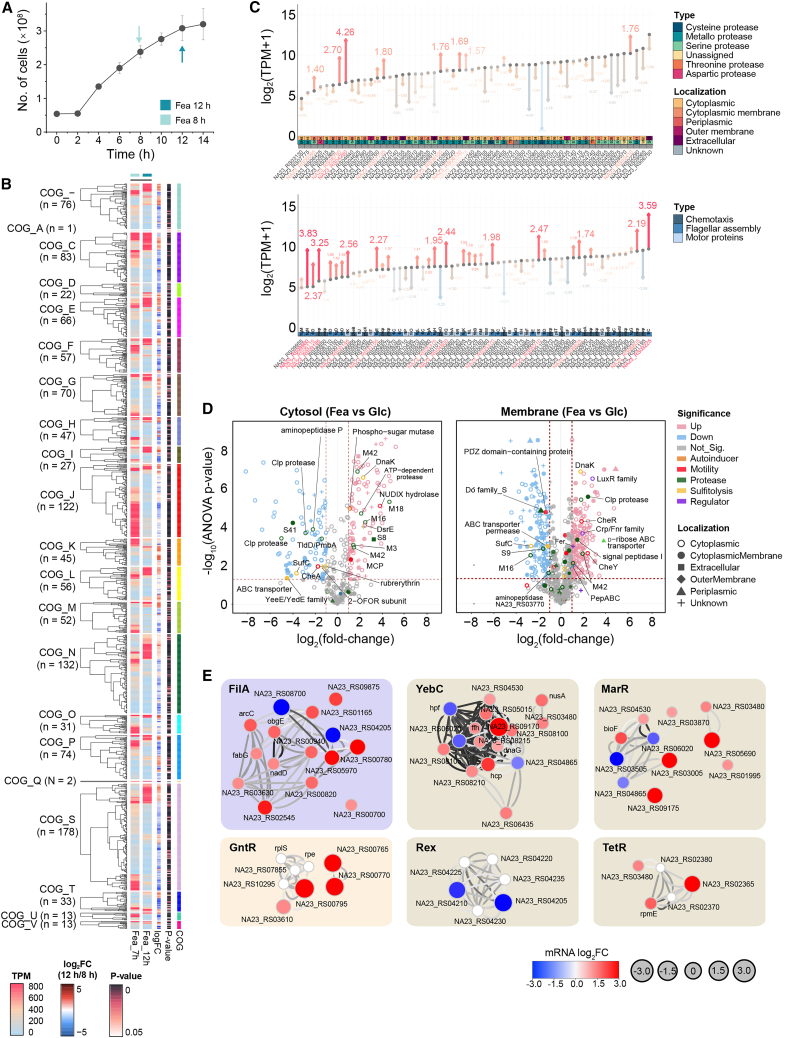
Temporal transcriptomic and proteomic profiling reveal a transition to sessile stress adaptation (A) Growth curve of Fea-grown cells showing RNA-Seq sampling points (8 h and 12 h). (B) Heatmaps showing time-resolved transcriptomic changes in feather-grown cells grouped by COG functional classes. (C) Differential expression of protease-related genes (top) and chemotaxis/motility genes (bottom) at 12 h relative to 8 h. (D) Volcano plots showing differentially expressed proteins (DEPs) in cytosolic (left) and membrane-associated (right) fractions from Fea-versus Glc-grown cells. (E) STRING-derived subnetworks of key transcriptional regulators (FliA, YebC, MarR, GntR, Rex, and TetR) controlling motility, proteostasis, and keratin metabolism. Nodes are colored by transcriptomic changes (log_2_FC, Fea vs. Glc).

To capture regulatory and functional transitions during keratin utilization, we compared feather-grown transcriptomes between early (8 h) and late exponential phase (12 h), integrated with quantitative proteomic profiling of cytosolic and membrane-associated fractions. This time window corresponds to the transition from active catabolism to a starvation-adaptive state. Transcriptomic analysis revealed selective reorganization of the proteolytic machinery. Of 57 predicted protease genes, ∼60% exhibited peak expression in feather-grown cells, with one S8-family protease exceeding 4,000 TPM (Figure S2C). Between 8 h and 12 h, stress-associated proteases (M42, M48, and S14) and protein quality-control enzymes (M15 and M38) were induced 3- to 8-fold, whereas most other protease transcripts declined (Figures 3A–3C and S3). This pattern suggests functional specialization of the protease repertoire during prolonged starvation. Concurrently, transcripts encoding chemotaxis regulators (CheA, CheC, and CheD) and sulfur/redox-associated proteins (glutaredoxin, cupin, rubredoxin, Fur, and TauE) were downregulated, indicating reduced environmental sensing and attenuated sulfitolytic activity as keratin hydrolysis progressed (Figures 3B, 3C, and S3; Data S1: Sheet 9). Notably, *safE*, encoding a sulfite exporter, was upregulated > 4-fold, potentially facilitating intracellular redox balance and localized extracellular disulfide reduction.

This transition provided a framework to link transcriptional reprogramming with corresponding changes in protein abundance, enabling assessment of how starvation-driven gene expression is translated into functional protein deployment during the motile-to-sessile switch. Proteomic profiling supported these transcriptional trends. Indeed, global translational capacity was markedly repressed, as evidenced by > 10-fold downregulation of ribosomal proteins and decreased expression of stringent-response associated GTPase (*obgE* and *hflX*), consistent with entry into a low-growth, energy-conserving state (Data S1: Sheet 9–12). In contrast, genes encoding cyclic-di-GMP turnover enzymes (EAL/GGDEF and HD-GYP domain proteins) increased 2- to 5-fold, consistent with regulatory shifts favoring sessile, biofilm-associated lifestyles (Figure S3). Feather-grown cells expressed more than twice as many membrane-associated proteins as glucose-grown cells (910 vs. 430), including metallopeptidases (M42 family), ABC transporters, and chemotaxis/biofilm regulators (CheR, Crp/Fnr family) (Figure 3D; Data S2). Cytosolic fractions were enriched in amino acid fermentation enzymes, glutamate metabolism, and gluconeogenesis, and stress-defense proteins (DnaK and GroES) along with redox regulators (Figures 3D and S4A). Notably, chemotaxis and flagellar proteins (CheR, CheY, CheB, FlhAF, and FliA) remained abundant in both cytosolic and membrane fractions, supporting a coordinated behavioral response coupling motility and surface colonization.

Consistently, several functional modules showing transcriptional retention during starvation, including stress-associated proteases, transport systems, and redox-related proteins, exhibited concordant enrichment at the protein level, particularly within membrane-associated fractions. This correspondence demonstrates that starvation-driven transcriptional changes are selectively stabilized and translated into functional protein assemblies supporting sessile persistence. Protein-protein interaction (PPI) network analysis revealed modular clusters corresponding to membrane proteolysis, flagellar assembly, sulfur and nitrogen metabolism, amino acid turnover, chemotaxis, ABC transporters, and redox-linked pathways (Figure S4B; Data S3). Regulatory hubs included FliA (motility), FadR (fatty acid metabolism), YebC (LPS and EPS synthesis), and MarR/TetR (AcrR) repressors, linking metabolic adaptation to behavioral and lifestyle transitions (Figures 3E and S4B).

Collectively, these data indicate a coordinated transcriptomic-proteomic transition in *F*. *islandicum* AW-1 from motility-associated growth toward a sessile, stress-tolerant, and persistence-like state. This transition involves selective protease retention, repression of energy-intensive processes, and activation of biofilm-associated regulatory circuits, enabling long-term survival on keratin substrates under soluble nutrient scarcity (Figure S4B).

### Metabolic remodeling links keratin-derived nutrient recycling with redox regulation

**Figure 4:**
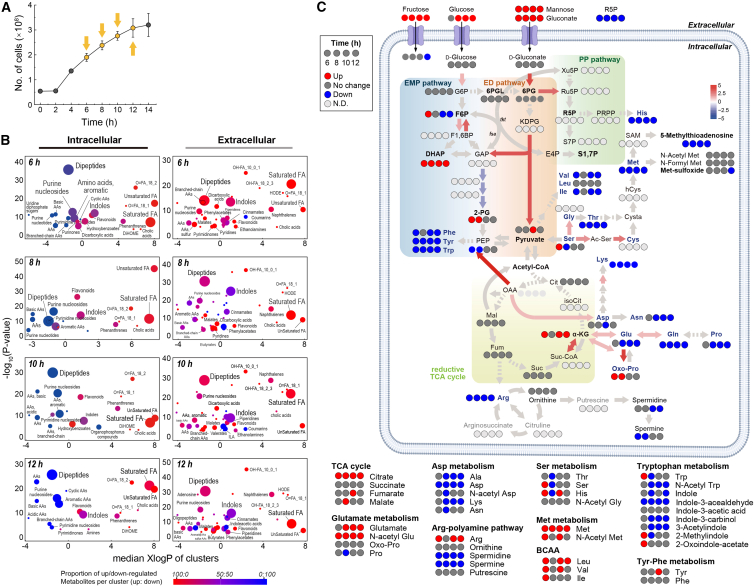
Temporal metabolomic shifts reveal metabolic remodeling during feather degradation (A) Growth curve with sampling points for metabolomic analysis (6–12 h). Data represent mean ± SD (*n* = 3). (B) Chemical enrichment analysis of metabolite clusters showing temporal shifts in intracellular (left) and extracellular (right) fractions. Bubble size indicates significance (−log_10_*p* value); color represents the proportion of upregulated versus downregulated metabolites in each cluster. (C) Mapping of metabolite changes (log_2_ fold-change) across central carbon metabolism and sulfur amino acid biosynthesis pathways. Metabolic rewiring toward the ED pathway, sulfur-containing amino acids (e.g., cysteine), and dipeptide accumulation highlights an adaptive strategy to conserve energy and maintain redox balance during starvation. Colored circles represent metabolites (red, increased; blue, decreased; gray, unchanged; black, undetected).

To assess metabolic adaptations during keratin utilization, we performed untargeted metabolomic profiling of intracellular and extracellular fractions from *F*. *islandicum* AW-1 cultures grown with or without native feathers, sampling at 6–12 h to capture key physiological transitions (Figure 4A). Across compartments, 323 intracellular and 335 extracellular metabolites were detected (Data S4). Principal component and partial least squares-discriminant analyses revealed clear separation between keratin-grown and control (mTF only) cultures in both fractions, indicating substantial metabolic remodeling (Figure S5A).

Amino acids, lipids, and redox-associated metabolites showed the most pronounced changes. Intracellular dipeptides (e.g., Ala-Ile, Gly-Val, and Ala-Leu) and free amino acids (e.g., Val, Ser, and Glu) declined progressively over time, while accumulating extracellularly, consistent with continuous proteolytic release from keratin substrates (Figures 4B, S5B, S5C, and S6–S8; Data S4). Sulfur-containing amino acids and derivatives, including cysteine and methionine, were enriched, in agreement with transcriptomic induction of sulfur assimilation and redox-related pathways. Lipid metabolism shifted toward both saturated and unsaturated fatty acids (e.g., palmitoleic acid, sphingosine, nervonic acid, and oleic acid), consistent with membrane remodeling and alternative energy utilization under nutrient stress. Chemical enrichment analysis indicated progressive depletion of dipeptides and purine nucleosides, late-stage accumulation of saturated fatty acids, and persistent enrichment of indoles and flavonoids-metabolite classes commonly associated with redox modulation and intracellular signaling processes (Figures 4B and S5–S8).

Structure similarity-based metabolite network analysis indicated coordinated clustering of sulfur amino acids, pyrimidine catabolites, and intermediates of the ED pathway (e.g., 6 PG, KDPG, etc.) and the TCA (succinate, α-KG, and malate), consistent with transcriptomic evidence for energy-efficient carbon metabolism under starvation (Figures 4C and S5C). Pathway enrichment analysis demonstrated pathway-specific and stage-dependent changes across a broad range of central carbon metabolism during feather utilization (Figure S9). Overall, glycolysis/gluconeogenesis showed significant enrichment across all stages. In contrast, the pentose phosphate pathway, pyruvate metabolism, and the TCA cycle showed relatively delayed enrichment, becoming significant primarily at later stages (10 and 12 h). Glyoxylate and dicarboxylate metabolism showed an increase in enrichment beginning at 8 h and remained significant thereafter, indicating early involvement of anaplerotic pathways during the transition toward stress-adaptive metabolism. Taken together, these patterns reflect a stage-dependent expansion of enriched pathways within central carbon metabolism. Notably, secondary metabolites such as indole-3-acetaldehyde and pseudouridine displayed dynamic temporal fluctuations, suggesting potential roles in stress-associated signaling and persistence-related regulation (Figure 4B; Data S4). These metabolic shifts are consistent with the coordinated transcriptional and proteomic reprogramming observed during the motile-to-sessile transition, reflecting downstream biochemical outcomes of starvation-driven keratin degradation.

Together, these data indicate that *F*. *islandicum* AW-1 engages in a starvation-adaptive metabolic program during keratin degradation, in which amino acid and lipid catabolism support nutrient recycling, sulfur-rich metabolites contribute to redox homeostasis, and ED-associated intermediates are consistent with energy conservation. This metabolic remodeling likely supports both individual cell- and coordinated community-level survival on keratin substrates under nutrient limitation. Taken together, these metabolomic patterns provide functional readouts of the upstream transcriptomic and proteomic adaptations, completing a multi-layered view of starvation-driven keratin utilization.

### The stringent response and cyclic-di-GMP signaling are linked to motility-biofilm transitions and persister-like adaptation

**Figure 5:**
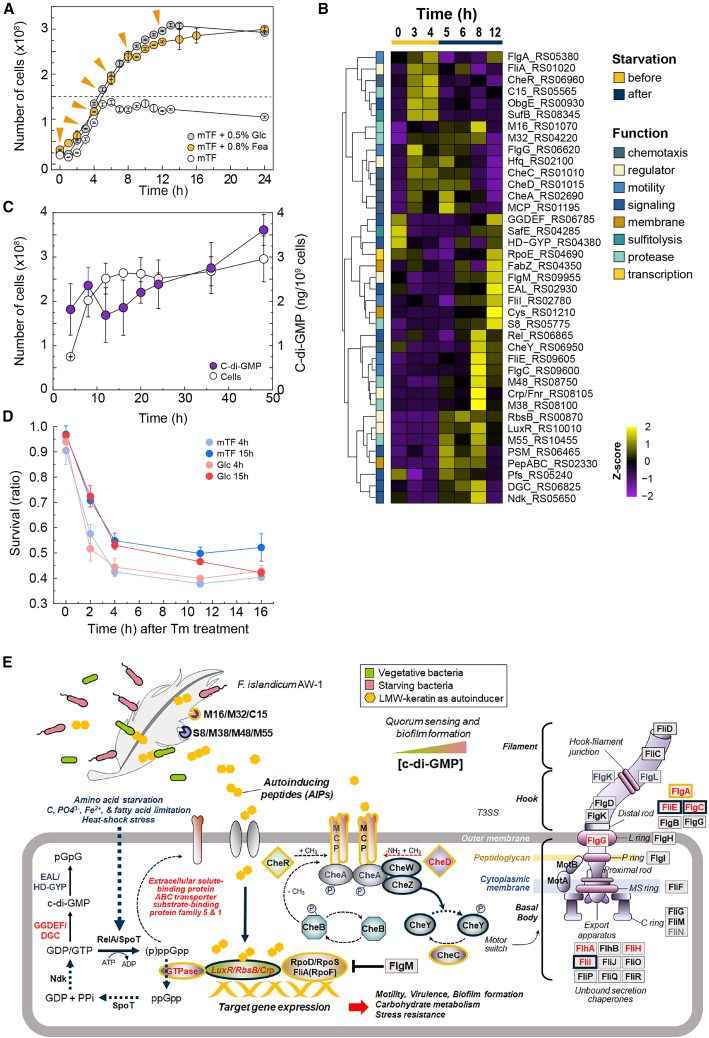
Stringent response and c-di-GMP signaling coordinate motility, biofilm formation, and persister-like adaptation (A) Growth curves in mTF medium supplemented with Glc, Fea, or no supplement, indicating sampling points for RT-qPCR. The dashed line denotes entry into the stationary phase in mTF only. (B) Heatmap of time-resolved expression (0–12 h) of genes associated with chemotaxis (blue), motility (green), sulfitolysis (yellow), proteolysis (purple), transcriptional regulation (orange), and membrane-associated factors (gray). *Z* scores indicate relative transcript abundance. (C) Quantification of intracellular c-di-GMP levels and viable cell counts over 48 h in Fea-supplemented medium (*n* = 3). (D) Survival curves following thiamphenicol treatment (200 μg/mL) in non-starved (mTF ± Glc, 4 h), starved (mTF + Glc, 15 h), and nutrient-depleted (mTF only, 15 h) cultures. (E) Integrated model summarizing regulatory networks activated under nutrient limitation. Starvation induces RelA/SpoT-mediated(p)ppGpp synthesis and GGDEF-domain DGCs driving cyclic-di-GMP accumulation. Keratin-derived peptides and sulfur metabolites modulate transcriptional regulators (e.g., LuxR, Crp, and RbsB), controlling motility, adhesion, and biofilm persistence in keratin-rich niches. Downstream effectors (e.g., FlgM and CheY) and Type III secretion systems (T3SS) components coordinate a reverse motile-to-sessile switch, enabling persistence in keratin-rich, nutrient-depleted environments.

To dissect regulatory dynamics during starvation-induced keratin degradation, we profiled time-resolved gene expression related to cyclic di-GMP (c-di-GMP) metabolism (*dgc*, GGDEF, and EAL), stringent response pathways (*rel* and *rpoE*), and biofilm-associated regulators (*luxR*), integrating these with c-di-GMP quantification and phenotypic assays (Figures 5A‒5C). Early during keratin utilization (6–8 h), genes encoding diguanylate cyclases (DGCs) (GGDEF-domain) and phosphodiesterases (EAL, HD-GYP domains) were upregulated 2–5 fold, coinciding with a measurable increase in intracellular c-di-GMP levels (Figures 5B and 5C). In parallel, the stringent response was transiently activated, as indicated by increased expression of RelA/SpoT homologs at 8 h, repression of ribosomal protein genes, and downregulation of anabolic pathways—signatures of growth arrest and energy conservation (Figure 5B: Data S1: Sheet 9).

During this interval, chemotaxis regulators (*cheA*, *cheR*, and *cheY*) and flagellar assembly genes (*fliA* and *fliC*) reached peak expression, consistent with enhanced motility during early substrate exploration and colonization (Figures 5B and 5C; Data S1: Sheets 1 and 9). By 12 h, c-di-GMP levels declined, motility-associated genes were repressed, and adhesion- and biofilm-associated genes were induced, marking a transition from motile to sessile lifestyles (Figures 5B and 5C). Confocal imaging confirmed the formation of microcolonies directly on feather surfaces (Figure 1H). Starvation also conferred antibiotic tolerance, a defining feature of persister-like states. Cells harvested from late-stationary glucose cultures (15 h) survived thiamphenicol exposure (200 μg/mL) more effectively than exponentially growing glucose-grown cells (4 h), displaying a biphasic killing profile characteristic of persistence (Figure 5D). This tolerance likely reflects the combined effects of stringent signaling, translational repression, and biofilm-associated protection.

Together, these observations are consistent with keratin degradation being embedded within a broader regulatory circuit, in which proteolytic release of peptides and sulfur-containing metabolites is coupled to intracellular signaling processes. Accumulated peptides may influence SpoT activity and c-di-GMP turnover, thereby synchronizing extracellular nutrient availability with transitions between motile and sessile states (Figures 5B and 5E). Membrane-associated proteases, particularly members of the S8 and M32 families, may contribute to this coupling by liberating peptide cues that link extracellular degradation to intracellular sensing (Figure 5E).

To evaluate the structural plausibility of this proposed peptide-linked feedback mechanism, we examined whether dipeptides accumulating during keratin utilization could be accommodated by candidate sensing and regulatory proteins. Using AlphaFold2-predicted structures and GNINA docking, the dipeptides Ala-Ile and Gly-Val exhibited energetically feasible binding to an methyl-accepting chemotaxis protein (MCP) sensory protein (NA23_RS01195; Vina −5.61/−5.18 kcal/mol; CNN pose 0.8995/0.8468), a Crp/Fnr-family transcriptional regulator (NA23_RS08105; Vina −6.01/−5.59 kcal/mol; CNN pose 0.6228/0.7353), and the D-ribose ABC transporter substrate-binding protein RbsB (NA23_RS00870; Vina −4.92/−4.44 kcal/mol; CNN pose 0.4447/0.9144) (Table S5; Figure S10). These structure-based analyses provide hypothesis-generating support for the feasibility that small peptides generated during keratin degradation can be accommodated by protein pockets associated with environmental sensing and transcriptional regulation.

Collectively, these findings are consistent with a starvation-adaptive network in *F*. *islandicum* AW-1 in which stringent response and c-di-GMP-associated signaling contribute to coordinated motility-biofilm transitions and promote persister-like survival in keratin-rich, nutrient-poor environments.

### Proposed model of starvation-induced keratin degradation and persistence in *F*. *islandicum* AW-1

**Figure 6:**
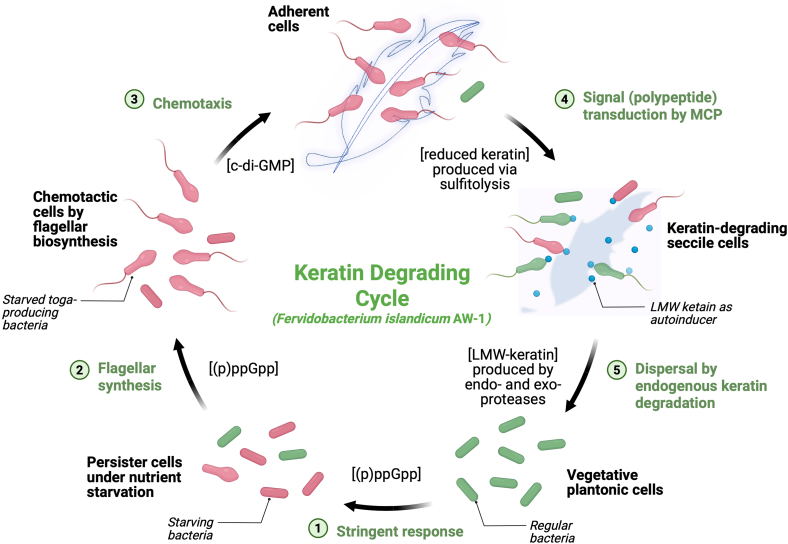
Proposed model for the starvation-adaptive keratin degradation cycle of F. islandicum AW-1 The cycle integrates regulatory, metabolic, and behavioral responses to nutrient limitation. (1) Starvation triggers stringent response signaling ([p]ppGpp) to arrest growth and promote stress adaptation. (2) Flagellar synthesis enables chemotaxis toward insoluble keratin substrates. (3) Cells adhere to keratin surfaces and initiate redox-mediated sulfitolysis to reduce disulfide bonds, enhancing protease accessibility. (4) Small keratin-derived peptides and sulfur metabolites act as signaling molecules sensed by MCPs and transcriptional regulators, stimulating biofilm-like sessile growth. (5) Membrane-bound S8/M48 proteases and cytosolic peptidases (M38 and C15) complete keratin degradation, providing nutrients for the population and facilitating dispersal. This integrated cycle promotes long-term persistence in keratin-rich, nutrient-poor environments.

Our integrated multi-omics analyses support a mechanistic model in which *F*. *islandicum* AW-1 couples keratin degradation to survival under nutrient scarcity (Figure 6). We propose that upon starvation, the bacterium engages membrane-associated proteolysis together with redox-mediated sulfitolysis to locally depolymerize keratin, resulting in the release of peptides and sulfur-containing metabolites. These products are subsequently imported via ABC transporters and metabolized through energy-efficient pathways, primarily the ED pathway and the reverse TCA cycle, while sulfur assimilation pathways contribute to maintaining intracellular redox balance.

At the regulatory level, stress-responsive networks involving the (p)ppGpp stringent response and c-di-GMP signaling are associated with a lifestyle transition from motile surface exploration to sessile biofilm formation. Sessile communities maintain close to the keratin substrate, enabling localized degradation and facilitating entry into a metabolically quiescent, persister-like state that confers stress tolerance. Accumulation of extracellular peptide and sulfur metabolites is proposed to provide feedback into these regulatory circuits, contributing to dynamic modulation of motility, adhesion, and metabolic activity.

Together, this starvation-responsive strategy integrates physical substrate colonization, surface-associated enzymatic disassembly of a highly recalcitrant protein polymer, metabolic rewiring, and stress adaptation. The model illustrates how an ancient extremophile leverages membrane-localized proteolysis and redox chemistry to persist in disulfide-rich, oligotrophic environments and provides a conceptual framework relevant to microbial ecology as well as biotechnological keratin valorization.

## Discussion

Keratin degradation has evolved independently across diverse microbial lineages, yet convergent evidence indicates that effective keratin utilization universally requires the coordinated disruption of disulfide crosslinks though sulfitolysis in combination with proteolytic cleavage of the peptide backbone.^26^ Although comparative studies of feather-degrading bacteria have traditionally emphasized the diversity of keratinases, growing evidence suggests that the regulation, localization, and redox coupling of sulfitolysis constitute a more decisive determinant of keratin-degrading capacity than protease repertoire alone.^16^^,^^21^ Our findings support this concept by demonstrating that in *F*. *islandicum* AW-1, keratinolytic proteases are constitutively expressed across various nutrient conditions, whereas efficient keratin degradation emerges specifically under starvation, when redox-mediated sulfitolysis and membrane-associated proteolysis become spatially and physiologically coordinated (Figures 1, 2, S1, and S2).

Our data indicate that keratin degradation in *F*. *islandicum* AW-1 is mediated by a functionally heterogeneous, cell-surface-associated protease system. The S8-family serine protease identified here is a close homolog of fervidolysin, whose crystal structure and endo-type proteolytic activity have been previously characterized.^27^ In addition, multiple metalloproteases from *F*. *islandicum* AW-1, including members of the M16, M32, M38, M55, and S16 families, have been biochemically validated in earlier studies for keratinolytic or proteolytic activity.^12^^,^^24^^,^^28^ Together, these prior biochemical data provide direct functional evidence that AW-1 possesses enzymatic machinery capable of cleaving the keratin peptide backbone. By contrast, the M48-family metalloprotease emerges from the present study as a starvation-responsive, membrane-associated candidate whose induction pattern and subcellular localization suggest a role in keratin processing or proteostasis maintenance under stress. However, its precise biochemical activity has not yet been experimentally validated. We therefore interpret the involvement of M48 proteases as a testable hypothesis generated by our integrated transcriptomic and proteomic analyses, rather than as a demonstrated catalytic function. Consistent with this model, diffusion barrier assays and microscopy demonstrate that keratin degradation requires direct cell-substrate contact, indicating that the process is not driven by diffusible extracellular enzymes or bulk chemical reductants. Instead, degradation relies on localized, surface-associated enzymatic activity coupled to cellular redox metabolism (Figure 1). This contact-dependent strategy distinguishes *F*. *islandicum* AW-1 from many aerobic keratin degraders and underscores the importance of physiological context in shaping degradative outcomes, particularly under anaerobic and oligotrophic conditions.

Sulfur metabolism represents a central adaptive axis enabling sulfitolysis during starvation.^12^^,^^21^ Unlike aerobic keratin degraders such as *Streptomyces* and *Bacillus* species, which generate sulfite from l-cysteine through oxygen-dependent pathways,^15^^,^^26^ the strict anaerobe *F*. *islandicum* AW-1 employs an alternative sulfur-based strategy. Starvation-induced activation of Suf (*sufCBDSU* and *tusA-yedE-dsr* operons) supports both Fe-S cluster biogenesis and redox processes linked to disulfide bond reduction, suggesting a strong coupling of sulfitolysis to metabolic flexibility.^12^^,^^21^ Although anaerobic sulfur metabolism could, in principle, generate diffusible sulfite, the contact-dependent degradation observed in two-chamber diffusion assays indicates that *F*. *islandicum* AW-1 preferentially relies on membrane-associated endoproteases (e.g., S8 family proteases) coupled with enzyme-mediated, NADPH-dependent reduction rather than bulk chemical sulfitolysis for efficient keratin breakdown (Figures 2 and 3).^12^^,^^21^

Importantly, the present work does not re-validate the biochemical basis of sulfur-dependent keratin degradation, which has been established in prior work, but instead extends these findings into a genome-scale, time-resolved framework. Our data demonstrates that sulfur metabolism is coordinately regulated with membrane-associated proteolysis, redox homeostasis, and metabolic rewiring during starvation-driven keratin utilization (Figures 3 and S4). The strict requirement for cell-substrate contact further supports a model in which sulfitolysis is spatially confined and functionally integrated with cell-surface enzymatic activity rather than mediated by diffusible reductants.

Beyond substrate depolymerization, keratin utilization in *F*. *islandicum* AW-1 is embedded within a broader starvation-adaptive program that integrates metabolic remodeling, regulatory signaling, and behavioral transitions (Figures 1, 2, 3, 4, 5, and 6). Rather than serving solely as a nutrient source, keratin degradation coincides with a shift toward energy-efficient carbon metabolism redox balancing, and repression of ATP-intensive biosynthetic processes (Figure 2). This metabolic state parallels adaptive strategies observed in diverse bacteria under prolonged nutrient stress^29^^,^^30^ and suggests that keratin functions not only as a resource but also as a physical and ecological scaffold supporting persistence-analogous behaviors. Notably, *F*. *islandicum* AW-1 exhibits multiple features commonly associated with bacterial persistence, including stringent response activation, translational repression, surface-associated aggregation, and increased antibiotic tolerance (Figures 2, 3, and 5).^31^ While persistence mechanisms remain incompletely understood even in mesophilic model organisms, conserved themes such as (p)ppGpp-mediated stringent responses,^1^^,^^32^ toxin-antitoxin systems,^33^ biofilm-associated dormancy,^34^ and community-level protection recur across phylogenetically distant bacteria.^32^^,^^35^^,^^36^ This regulatory architecture enables dynamic adjustment between exploration and exploitation under fluctuating nutrient availability and mirrors regulatory logics observed in both environmental and pathogenic bacteria, albeit implemented here in the context of an extreme thermophilic lifestyle. The presence of such conserved stress-response features in a deeply branching hyperthermophilic anaerobe suggests that persistence-like strategies may represent ancient survival solutions that predate the diversification of modern bacterial lineages.^37^

At the regulatory level, this multifaceted response appears to operate through a network involving stringent-response-associated transcriptional regulators (TetR and MarR), motility and chemotaxis factors (CheR and FliA), and membrane-remodeling genes (Figures 2, 3, and 4) that collectively coordinate substrate localization, colonization, nutrient scavenging, and stress mitigation. This framework also includes functionally uncharacterized proteases such as S14, and M48, co-expressed with cytosolic peptidases (M38 and C15) (Figures 2 and 3), which likely contribute to integrating keratin processing with proteostasis maintenance under extreme conditions.^28^^,^^38^^,^^39^

Behaviorally, starved *F*. *islandicum* AW-1 appears to initially minimize energy expenditure while maintaining motile exploration until stochastic adhesion to keratin surfaces occurs. This encounter is followed by degradation-product-associated signaling that promotes a transition from motile colonization to sessile, biofilm-like growth, enabling community-level keratin degradation directly on substrate surfaces (Figures 1H, 5E, and 6). This lifestyle shift enhances substrate access, spatial niche establishment, and survival during prolonged nutrient stress.^40^^,^^41^^,^^42^^,^^43^ Early activation of flagella and chemotaxis genes (e.g., *fliC* and *flhA*) facilitates substrate targeting, while increasing c-di-GMP levels repress motility and induce sessility (Figures 2, 3C, 5B, and 5C). Oscillation in c-di-GMP thus appear to function as a regulatory switch enabling dynamic control over environmental sensing, adhesion, and persistence on keratin substrates^44^ (Figure 6). This resulting sessile state lacks canonical EPS components, suggesting alternative adhesion mechanisms mediated by low-molecular-weight keratin-derived peptides and proteinaceous surface structure such as the Thermotogales-specific toga.^45^^,^^46^ Co-expression of peptide transporters, carboxypeptidases (e.g., M55, M32, and M16), and MCPs further supports a quorum-like signaling framework coordinating community-level behavior such as persistence and biofilm maturation (Figure 5E).^47^^,^^48^

Metabolomics analyses reinforce this integrated model by revealing dynamic accumulation of dipeptides, amino acids, fatty acids, and sulfur metabolites during keratin utilization (Figures 4 and S5). These metabolites likely serve both nutritional and signaling roles. RelA/SpoT homolog (RSH)-mediated stringent responses are canonically regulated by amino acid starvation (resulting in the accumulation of uncharged tRNAs) and broader nutrient cues like carbon, fatty acid, and nitrogen starvation.^49^^,^^50^ Branched-chain amino acids (leucine, isoleucine, valine) have been shown to modulate Rel-type RSH proteins via ACT (aspartokinase, chorismite mutase, TyrA) domain, adjusting (p)ppGpp hydrolase activity in response to external nutritional signals.^51^ Indole derivatives, such as the accumulated indole-3-acetaldehyde, acts as a key signaling molecule regulating biofilm formation and quorum sensing, albeit its role is variable dependent on species and concentration.^52^^,^^53^^,^^54^ Additionally, the accumulated dipeptides may serve as chemotactic signals sensed by MCPs (Figure S7), as peptide-mediated chemotaxis has been reported in diverse bacteria including *E*. *coli* and *Pseudomonas* species.^55^^,^^56^ Together with upregulation of the ED pathway and reductive TCA cycle (via ATP citrate lyase and 2-oxoglutarate; ferredoxin oxidoreductase), these profiles indicate a metabolic shift favoring NADPH generation as a cofactor for reductases and biosynthetic precursor supply with minimal ATP expenditure, consistent with a starvation-adaptive regulatory state (Figure 2).

Collectively, these findings establish a model in which *F*. *islandicum* AW-1 leverages keratin degradation as an integrated starvation survival strategy (Figure 6). Membrane-associated proteolysis and redox-mediated sulfitolysis enable localized access to a highly recalcitrant substrate, while interconnected metabolic and regulatory networks coordinate energy conservation, lifestyle transitions, and persister-like tolerance. This strategy parallels those employed by diverse environmental and clinical biofilm formers^40^^,^^57^^,^^58^^,^^59^ but is uniquely adapted to the disulfide-rich, anaerobic, and oligotrophic niches of Thermotogales extremophiles.^60^

From a biotechnological perspective, the ability of *F*. *islandicum* AW-1 to efficiently depolymerize native keratin under extreme conditions highlights its potential for sustainable biomass valorization. Coordinated release of peptides, amino acids, lipids, and redox-active compounds could be exploited for value-added product recovery, biofertilizer development, and extremophile-based industrial bioprocessing.^61^ More broadly, this work provides a systems-level framework for understanding how ancient extremophiles integrate surface-associated degradation, metabolic flexibility, and stress adaptation to endure nutrient-poor environments.

### Limitations of the study

While this study provides an integrated multi-omics framework for understanding starvation-driven keratin degradation in *F*. *islandicum* AW-1, several limitations constrain direct mechanistic interpretation. First, the lack of genome-editing and genetic perturbation tools for this organism precludes direct functional validation of candidate genes implicated in keratin degradation, stress adaptation, and regulatory signaling. Although multiple *F*. *islandicum* AW-1 proteases, including S8-family serine proteases and several metalloproteases, have been biochemically characterized in previous studies,^12^^,^^24^^,^^27^^,^^28^ not all proteases highlighted by the present systems-level analysis have been functionally validated. In particular, the proposed role of the M48-family metalloprotease is currently supported by expression dynamics and subcellular localization rather than direct biochemical evidence. Ongoing cloning and enzymatic characterization will be required to define its precise function.

Second, the roles of sulfur metabolism and Fe-S cluster biogenesis in keratin degradation have been established in earlier biochemical and physiological studies.^12^^,^^21^ In the present work, these processes are examined at the genome-scale as part of an integrated starvation-adaptive program. However, the absence of genetic tools for *F*. *islandicum* AW-1 prevents targeted perturbation experiments (e.g., gene knockouts) to dissect causal regulatory relationships within this network.

Third, limited analytical resolution prevented simultaneous, time-resolved quantification of key regulatory metabolites—most notably (p)ppGpp alongside c-di-GMP—thereby restricting direct assessment of their temporal interplay. In addition, although differential expression analyses identified putative transcriptional regulators associated with keratinolysis and stress responses, we were unable to perform binding motif validation or regulatory perturbation experiments (e.g., ChIP sequencing or targeted knockdowns). Consequently, regulatory inferences rely on sequence homology, operon context, and co-expression patterns rather than direct evidence of transcription factor-DNA interactions.

Finally, while integrated transcriptomic and metabolomic signatures are consistent with altered stringent response- and c-di-GMP-associated regulatory programs, and structure-based docking analyses support the structural plausibility of dipeptide accommodation by candidate sensing or regulatory proteins, these observations remain correlative and computational. Direct biochemical assays (e.g., binding/competition, reporter readouts), together with genetic perturbation, will be required to establish causal signaling relationships. Future work will therefore focus on developing genetic tools for *F*. *islandicum* AW-1 and applying higher-resolution, time-resolved metabolic and signaling analyses to identify bona fide signaling molecules and define their mechanistic roles in starvation-driven keratin degradation.

## Resource availability

### Lead contact

Further information and requests for resources and reagents should be directed to and will be fulfilled by the lead contact, Dong-Woo Lee (leehicam@yonsei.ac.kr).

### Materials availability

This study did not generate new unique reagents. The *F*. *islandicum* AW-1 strain is subject to patent restrictions, and therefore, cannot be provided, even upon request.

### Data and code availability

• The mass spectrometry proteomics data have been deposited in the ProteomeXchange Consortium via the PRIDE partner repository with the dataset identifier PXD027063.
• RNA-Seq data have been deposited in the NCBI Sequence Read Archive (SRA) under accession numbers SRR33382689–SRR33382697 and SRX219354-7.
• The metabolomics raw data have been deposited in the MassIVE repository (https://massive.ucsd.edu) under accession ID MSV000099151. It is recommended to use a preferred FTP client program (e.g., FileZilla, WinSCP, CoreFTP, or CoffeeCup Free FTP).
• This article does not report original code.
• Any additional information required to reanalyze the data reported in this article is available from the lead contact upon request.

## Acknowledgments

This work was supported by the 10.13039/501100003725National Research Foundation (NRF) of Korea grant (RS-2023-NR076895 to D.-W.L.), funded by the 10.13039/501100014188Ministry of Science and ICT (MSIT), Republic of Korea. This work was also partly supported by the NRF of Korea (grants RS-2025-02215093 to D.-W.L. and RS-2022-NR068419 to D.Y.L). We thank Jae-Won La for their technical support during the cultivation of cells and preparation of subcellular fractionated samples for keratinolytic assay and metabolomic analysis.

## Author contributions

J.-Y.S., J.-Y.K., H.-S.J., J.-H.B., N.E.K., H.H.S., S.-H.B., Y.-J.L., B.-C.K., D.Y.L., and D.-W.L. formulated the research plan, carried out experiments, analyzed and interpreted the data, and drafted the manuscript. J.-Y.S., J.-Y.K., H.-S.J., D.Y.L., and D.-W.L. participated in the design of the study and analyzed and interpreted the data. D.Y.L. and D.-W.L. conceived, planned, and supervised the study.

## Declaration of interests

The authors declare no competing financial interests.

## STAR★Methods

### Key resources table

**Table:** 

REAGENT or RESOURCE	SOURCE	IDENTIFIER
**Chemicals, peptides, and recombinant proteins**		

SYTO9	Invitrogen, MA, USA	Cat# S34854
Propidium iodide	Invitrogen, MA, USA	Cat# P1304MP
CPM (7-diethylamino-3-(4’-maleimidylphenyl)-4-methyl-coumarin)	Invitrogen, MA, USA	Cat# D346
iScript cDNA Synthesis Kit	Bio-Rad, CA, USA	Cat# 1708890
2× SsoAdvanced™ Universal SYBR Green Supermix	Bio-Rad, CA, USA	Cat# 1725271
Thiamphenicol	Sigma-Aldrich, MO, USA	Cat# T0261

**Critical commercial assays**		

RNeasy Mini Kit	QIAGEN, Hilden, Germany	Cat# 74104
TruSeq RNA Sample Prep Kit v2	Illumina, CA, USA	Cat# RS-122
TruSeq DNA Nano Prep Kit	Illumina, CA, USA	Cat# 20015964
c-di-GMP ELISA kit	EIAab Science, Wuhan, China	Cat# E0152Ge

**Deposited data**		

RNA-seq data	This study	SRR33382689–SRR33382697 and SRX219354-SRX219357
Proteomics data	This study	PXD027063
Metabolomics data	This study	MSV000099151

**Experimental models: Organisms/strains**		

*F. islandicum* AW-1	Yonsei University	Korean Culture Collections of Microorganisms (KCCM) 80008

**Oligonucleotides**		

See Table S4 for oligonucleotides used in this study.	This study	N/A

**Software and algorithms**		

STRING v11.5	Szklarczyk D et al. 2023	https://string-db.org
Cytoscape v3.8.2 and v3.7.2	Shannon P et al. 2003	https://cytoscape.org/
FastQC v0.11.8	Andrews S	https://github.com/s-andrews/FastQC
Trimmomatic v0.39	Bolger AM et al. 2014	http://www.usadellab.org/cms/?page=trimmomatic
Bowtie2 v2.4.4	Langmead B et al. 2012	https://github.com/BenLangmead/bowtie2
featureCounts v2.0.1	Liao Y et al. 2014	https://subread.sourceforge.net
edgeR v3.40.2	Robinson et al. 2010	https://bioconductor.org/packages/release/bioc/html/edgeR.html
DNMB	Sung et al. 2026	https://github.com/JAEYOONSUNG/DNMB
InterProScan	Philip J et al. 2014	https://github.com/ebi-pf-team/interproscan
ProteinPilot v5.0	SCIEX, Ontario, Canada	https://sciex.com/products/software/proteinpilot-software
ProteoWizard	MC Chambers et al. 2012	https://proteowizard.sourceforge.io
Perseus™	Tyanova et al. 2016	https://maxquant.net/perseus/
MS-DIAL v5.5.250221	Tsugawa H et al. 2015	https://github.com/systemsomicslab/MsdialWorkbench
SIMCA 15	Umetrics AB, Umea, Sweden	https://shop.sartorius.com/ww/p/simca/M_SIMCA
Origin 2025b	OriginLab, MA, USA	https://www.originlab.com/2025
GraphPad Prism 7	GraphPad Software Inc., CA, USA	https://www.graphpad.com
ChemRICH	DK Barupal et al. 2017	https://chemrich.fiehnlab.ucdavis.edu
MetaboAnalyst v6.0	Z Pang et al. 2024	https://www.metaboanalyst.ca

**Other**		

HPLC Waters 2695 Alliance	Waters, USA	Cat# WAT270008
Aminex HPX-87H column	Bio-Rad, CA, USA	Cat# 1250140
Scanning electron microscopy (SEM) SU8220	Hitachi, Tokyo, Japan	Cat# SU8220
Transmission electron microscopy (TEM) HT7700	Hitachi, Tokyo, Japan	Cat# HT7700
Confocal laser scanning microscopy LSM 700	Carl Zeiss, Oberkochen, Germany	Cat# 12114
Amino acid analyzer L-8900	Hitachi, Tokyo, Japan	Cat# L8900AAA
Two chamber system	DWK Life Science, Wertheim, Germany	N/A
Illumina HiSeq 2500 platform	Illumina, CA, USA	Cat# SY-401-2501
Illumina NovaSeq 6000 platform	Illumina, CA, USA	Cat# 20012850
Triple-TOF 5600+ Mass Spectrometer	SCIEX, Ontario, Canada	Cat# TY-82363
NanoFlex cHiPLC	Eksigent Technologies, CA, USA	Cat# 950-00070
Q-Exactive Plus Orbitrap	Thermo Fisher Scientific, MA, USA	Cat# IQLAAEGAAPFALGMBDK
Ultimate-3000 UPLC	Thermo Fisher Scientific, MA, USA	Cat# 5082.0040
ACQUITY UPLC BEH C18 column	Waters, MA, USA	Cat# 186002350
CFX Connect™ Real-Time System	Bio-Rad	Cat# 185-5201

### Experimental model and study participant details

The *F. islandicum* AW-1 was isolated from a geothermal hot stream in Indonesia.^20^ In this study, the strain was used as the model organism for keratin degradation analysis. Cells were cultured under anaerobic conditions at 70°C in mTF, as described in the method details section. This study did not involve human or animal subjects; therefore, sex and gender considerations are not applicable.

No cell lines were used in this study.

No human subjects or samples were involved in this study.

### Method details

#### Bacterial strains and growth conditions

*Fervidobacterium islandicum* AW-1 was grown anaerobically in modified *Thermotoga*-*Fervidobacterium* (mTF) medium^62^ at 70°C and pH 7.0, supplemented with various carbon/nitrogen sources, as previously described.^20^ For feather-based cultures, mTF medium contained 8 g of native chicken feathers, 1 g of yeast extract, 1.6 g of K_2_HPO_4_, 0.8 g of NaH_2_PO_4_·H_2_O, 0.16 g of MgSO_4_·7H_2_O, 0.1 g of NH_4_Cl, 10 mL of a vitamin solution,^63^ 10 mL of a trace element stock solution,^64^ 3 mL of 25 % (w/v) Na_2_S⋅9H_2_O (pH 7.0), and 1 mL of 0.1 % (w/v) resazurin per liter.^64^ Media were boiled for 20 min, N_2_-flushed, reduced with Na_2_S·9H_2_O (pH 7.0), and autoclaved at 121°C for 20 min. Cells were maintained in 6% (v/v) dimethyl sulfoxide (DMSO) at -80°C.

#### Growth characterization and nutrient conditions

To assess growth under alternative nutrient conditions, mTF medium was supplemented with 5 g/L glucose, peptone, tryptone, or casein, in place of 8 g/L feathers. Seed cultures (50 mL) were pre-grown in glucose-supplemented mTF medium for 10 h at 70°C. Growth was monitored by direct cell counting using a Neubauer chamber (Marienfeld, Germany) and phase-contrast microscopy (DM750, Leica, Germany). Optical density was also measured at 600 nm (Ultrospec 8000, GE Healthcare, UK).

#### Feather degradation and organic acid analysis

Feather degradation was evaluated by filtering culture broth through No. 20 filter paper, followed by drying residual feathers at 70°C overnight. Organic acid profiles were determined by HPLC (Waters 2695 Alliance, USA) using an Aminex HPX-87H column with 5 mM H_2_SO_4_ as mobile phase (35°C, 0.45 mL/min). Metabolites were detected by UV and RI detectors. Retention times were as follows: oxalic acid (9.22 min), citric acid (11.03), malic acid (13.18), pyruvic acid (14.82), succinic acid (16.20), lactic acid (17.68), alanine (18.18), formic acid (18.78), acetic acid (20.52), and ethanol (28.35).

#### Electron and confocal microscopy

For transmission electron microscopy (TEM) and scanning electron microscopy (SEM), cells were harvested by centrifugation (10,000 × g, 10 min, 4°C), fixed in 2.5% (w/v) glutaraldehyde in 0.1 M Na_2_HPO_4_/KH_2_PO_4_ buffer (pH 7.2) at 4°C, and post-fixed with 1% (w/v) OsO_4_. Samples were dehydrated through a graded ethanol series (50 to 100%). For TEM, samples were embedded, sectioned (EM UC7/FC7, Leica), stained with 1% (w/v) ammonium molybdate, and imaged using a Hitachi HT 7700 at 80 kV. For SEM, freeze-dried samples were sputter-coated with platinum and imaged using an SU8220 (Hitachi, Japan) SEM at 15 kV.

For confocal laser scanning microscopy (CLSM), cells were stained with SYTO9 (8 μM) and propidium iodide (PI, 50 μM) and imaged using an LSM 700 confocal microscope (Carl Zeiss). Feather cysteine residues were fluorescently labeled with CPM dye (7-diethylamino-3-(4’-maleimidylphenyl)-4-methyl-coumarin) at a 10:1 dye: thiol molar ratio, followed by 2 h incubation at room temperature. Excitation wavelengths were 480 nm (SYTO9), 538 nm (PI), and 375 nm (CPM).

#### Subcellular fractionation and protease assays

Cells grown on proteinaceous substrates or feathers were pelleted by centrifugation (10,000 × g, 30 min, 4°C) and disrupted by sonication on ice. Crude lysates were centrifuged at 15,000 × g (30 min, 4°C), and the supernatant was ultra-centrifuged at 100,000 × g (2 h, 4°C) to separate cytosolic (supernatant) and membrane (pellet) fractions. Membrane proteins were solubilized in 50 mM Tris-HCl (pH 7.5) with *β*-dodecylmaltoside (1:1, w/w) on ice for 30 min and ultracentrifuged at 100,000 × g (30 min, 4°C) to obtain the solubilized membrane protein fraction.

Free amino acids were quantified by phenylisothiocyanate (PITC) derivatization and analyzed on a L-8900 amino acid analyzer (Hitachi, Japan) or by ninhydrin assay.^65^ Protease activity was determined using 0.2% (w/v) casein (Sigma) as substrate, following a modified Kunitz method.^66^ The reaction mixture (500 μL) contained 50 μg of enzyme and 0.2% (w/v) casein in 50 mM potassium phosphate buffer (pH 7.0). After incubation at 90°C for 20 min, the reaction was terminated with 5% (w/v) trichloroacetic acid (TCA), followed by centrifugation (10,000 × g, 30 min, 4°C). The absorbance of the supernatant was measured at 280 nm. One unit (U) of protease activity was defined as the enzyme amount increasing absorbance at 280 nm of 0.01 per min.

Keratinolytic activity was measured on native feathers or soluble keratin (Chr. 2, 0.2%).^25^ Reaction mixtures (5 mL) containing 0.5 mg of crude extract, 50 mM potassium phosphate buffer (pH 7.0), and 10 mM dithiothreitol (DTT) were incubated anaerobically at 75°C. One unit (U) of keratinase activity was defined as the amount of enzyme required to produce 1 μmol of product per min.

#### Two-chamber diffusion system

An anaerobic system was constructed using two Wheaton® serum bottles (223747, DWK Life Sciences) separated by a 0.20 μm cellulose nitrate membrane (Whatman), sealed with rubber gaskets and a 64 mm ball joint clamp (SM.28633500, SciLab). Chambers were pre-filled with 0.8% (w/v) feather-containing water and mTF medium, autoclaved, and equilibrated anaerobically. Manual sterile venting prevented pressure imbalances.

#### Transcriptome analysis

Mid-exponential cells grown in mTF medium with glucose, peptone, tryptone, or feathers were harvested, flash-frozen, and stored at -80°C. Total RNA was extracted using the RNeasy Mini Kit (QIAGEN) with on-column RNase-free DNase I treatment. RNA quality was verified using RNA electropherograms (Agilent 2100 Bioanalyzer) and RNA integrity numbers (RIN) scores.^67^

For RNA-Seq, total RNA (10 μg) was used to construct sequencing libraries. mRNA was enriched using Dynabeads (Life Technologies, USA), and cDNA libraries were prepared using the TruSeq RNA Sample Prep Kit v2 (Illumina, USA). Paired-end 150-bp reads were generated on the NovaSeq 6000 platform and mapped to the *F. islandicum* AW-1 genome (GenBank: NZ_CP014334.1) using Bowtie2 (v2.4.4) with default parameters. Gene-level read quantification was performed using featureCounts (v2.0.1).^68^ Raw read counts were normalized to transcripts per kilobase million (TPM),^69^ and differential gene expression was assessed using a exact test based on TPM values implemented in the *edgeR* package.^70^ RNA-Seq data have been deposited in the NCBI Sequence Read Archive (SRA) under accession numbers SRR33382689–SRR33382697 and SRX219354-7. Summary read statistics are in Table S3. On average, ∼95% of predicted ORFs were transcribed under all tested conditions. The complete dataset is available as Data S1.

#### Proteomics

Proteins were extracted and digested using the Filter-Aided Sample Preparation (FASP) method.^71^

Cells were lysed in SDT buffer (4% SDS, 100 mM Tris/HCl pH 7.6, 0.1 M DTT, 1 mM EDTA, 1× protease inhibitor cocktail), denatured at 95°C for 10 min, and centrifuged at 16,000 × g for 5 min. Supernatants were processed on Micron YM-30 filters with UA buffer (8 M urea in 0.1 M Tris/HCl pH 8.5) to remove detergent. Alkylation was performed with 50 mM iodoacetamide, followed by buffer exchange with 50 mM ammonium bicarbonate. Proteins were digested on-filter with trypsin (1: 50, w/w) overnight at 37°C. Peptides were recovered by centrifugation, acidified with 1% formic acid, and stored at -20°C until analysis.

Proteomic analysis was performed on a Triple-TOF™ 5600+ Mass Spectrometer (Sciex) coupled to an Eksigent NanoFlex cHiPLC system.^72^ Peptides were loaded onto a C18 trap column (0.5 mm × 200 μm) at 1 μL/min and separated on an analytical C18 column using a 90-min linear gradient (2–35% acetonitrile, 0.1% formic acid) at 400 nL/min. For data-dependent acquisition (DDA), survey scans (250 ms) were followed by MS/MS scans (150 ms) of the top 20 ions (precursor intensity >135, charge >1, exclusion 15 s). SWATH-MS was performed using variable 20 Da/mass windows (1 Da overlap) over 400–1,000 m/z, with dynamically adjusted collision energy (±15 eV spread) and 100 ms accumulation per scan (total duty cycle of 3.1 s in high-sensitivity mode).

Raw MS data were analyzed with ProteinPilot (V5.0, SCIEX) against the 8460.FIAW1.2.CDS-Protein_MaxQuant_Contaminants database (2,404 entries), with trypsin specificity. ProteinPilot Software was searched with a fragment ion mass tolerance of 0.100 Da and a parent ion tolerance of 0.050 Da, applying fixed modifications for cysteine (+ 57 Da for carbamidomethylation) and biological modifications/artifacts such as methionine oxidation (+ 16 Da). Peptide and protein FDRs were controlled at <1%. SWATH-MS data (wiff files) were converted to mz5 format using ProteoWizard software and analyzed in Skyline, with three isotopic peaks per precursor and fragment, 20 ppm mass accuracy, and a 10 min retention window. Peak identity was verified by co-evolution profiles and spectral similarity. Processed data were visualized and statistically analyzed using Perseus™ software, including principal component analysis (PCA) and clustering. Proteomics data are available via ProteomeXchange (PXD027063).

#### Protein interaction network analysis

STRING (ver. 11.5) interaction data for organism #2423 (*F. islandicum*) were manually curated and updated based on domain architecture and KEGG pathway mapping via InterProScan (Data S3). Networks were visualized in Cytoscape (ver.3.8.2.).

#### Metabolomics

Cells (∼1.0 × 10^9^) were harvested by centrifugation (10,000 × *g*, 5 min), lyophilized, and disrupted using a stainless-steel ball (5 mm) in a Mixer Mill MM400 (Retsch GmbH & Co., Germany). Intracellular metabolites were extracted in methanol:isopropanol: water (3:3:2, v/v/v, 1500 μl), sonicated (5 min), and centrifuged (16,100 rcf, 5 min, 4°C). Supernatants were dried using a speed vacuum concentrator. For extracellular profiling, culture media were dried and extracted using the same solvent.

Dried extracts were reconstituted in 80% methanol (50 μL) and analyzed on a Q-Exactive Plus Orbitrap (Thermo Fisher Scientific, USA) coupled to an Ultimate-3000 UPLC system with a BEH C18 column (2.1 × 100 mm, 1.7 μm, Waters). Mobile phases were water and acetonitrile with 0.1% formic acid (positive mode) or 0.1% acetic acid (negative mode). The 15 min gradient started at 0.5% organic for 0.1 min, ramped to 80% over 10 min, increased to 99.5% by 10.1 min, held to 12 min, then re-equilibrated. Full-scan spectra were collected from 80–1200 Da (MS resolution 70,000; dd-MS2 spectra resolution 17,500) with stepped collision energies (30, 40, and 50 eV) and a scan range of m/z 200–2000. The maximum injection time was set to 100 ms with an automatic gain control (AGC) of 10e6 charges. Data were processed using MS-DIAL (version 5.5.250221) with mass tolerances of 0.005 Da for MS1 and 0.01 Da for MS2, and retention time alignment tolerance of 0.5 min.

Statistical analyses included principal component analysis (PCA) and partial least squares-discriminant analysis (PLS-DA) using SIMCA 15 (Umetrics AB, Sweden). Chemical enrichment analysis was performed using the ChemRICH program, and volcano plots were generated with GraphPad Prism 7 (GraphPad Software Inc., USA). Metabolic network visualization was based on structural similarity (Tanimoto score) using Cytoscape (v3.7.2). Multivariate empirical Bayes statistical time-series analysis (MEBA) was performed using the time-series analysis tool, web-based easy-to-use platform, MetaboAnalyst. Metabolic pathway analyses were performed on samples collected at 6, 8, 10, and 12 h using the Pathway Analysis module of MetaboAnalyst 6.0 based on the KEGG pathway library. The resulting -log_10_(P-value) and impact scores were integrated across time points and visualized using R packages to illustrate these dynamic metabolic reorganizations.

The metabolomics raw data has been deposited in the MassIVE repository (https://massive.ucsd.edu) under accession ID MSV000099151. It is recommended to use a preferred FTP client program (e.g., FileZilla, WinSCP, CoreFTP, or CoffeeCup Free FTP).

#### Profiling of dynamic expression genes using qRT-PCR

For dynamic gene expression profiling, cDNA was synthesized from *F. islandicum* AW-1 cultures harvested at 3, 4, 5, 6, 8, and 12 hours post-inoculation, as previously described.^12^ All cDNA samples were diluted to 10 ng/μL prior to amplification. Quantitative real-time PCR (qRT-PCR) reactions (20 μL total volume) were prepared using 2× SsoAdvanced™ Universal SYBR Green Supermix (Bio-Rad), containing SYBR Green I, with 20 ng cDNA and 250 nM of each primer. Amplification was conducted on a CFX Connect™ Real-Time System (Bio-Rad) under the following cycling conditions: 95°C for 30 s, followed by 40 cycles of 95°C for 10 s and 58°C for 30 s. Primer sequences are listed in Table S4. Standard curves were generated from serial dilutions of pooled cDNA to assess amplification efficiency, following the MIQE guidelines.^73^ Expression levels were normalized to the *rpo*D gene (encoding the σ^70^ RNA polymerase subunit) as the internal reference.

#### Quantification of cyclic-di-GMP

Intracellular cyclic-di-GMP (c-di-GMP) levels were measured in *F. islandicum* AW-1 cultured anaerobically in mTF medium with 0.8% feathers. Samples were collected every 4 h up to 24 h and every 12 h thereafter up to 48 h. To eliminate feather debris, cultures were filtered through Whatman® Puradisc 13 syringe filters (5.0 μm, PTFE; Cytiva, USA). Cells were washed with PBS (pH 7.0), and OD600 values were measured to estimate cell density based on a previously established standard curve (6.2E+08 × (Abs.) + 2.7E+07).

Intracellular c-di-GMP was extracted using a modified heat and ethanol precipitation method, as previously described.^74^ For extraction, 1 × 10^9^ cells were fixed with 0.19% formaldehyde on ice for 10 min, washed with PBS (pH 7.0), then boiled in distilled water (100°C, 10 min). After cooling on the ice, cell lysates were mixed with ice-cold ethanol (35:65, v/v), centrifuged at 16,000 × g for 10 min at 4°C, and supernatants were pooled in three extraction rounds. Samples were flash-frozen in liquid nitrogen and lyophilized. Intracellular c-di-GMP was quantified using a commercial ELISA kit (EIAab) according to the manufacturer’s instructions.

#### Observation of antibiotic tolerance

To evaluate the antibiotic tolerance of starved versus non-starved cells, *F. islandicum* AW-1 was cultivated in mTF medium with or without 0.5% (w/v) d-glucose supplementation. Cells were harvested at time points corresponding to the exponential phase (4 h), late exponential/early stationary phase (10–12 h), and late stationary/early death phase (15–16 h) based on the mTF culture growth curve. After harvest, cell densities were normalized based on OD_600nm_ measurements and resuspended in fresh mTF medium containing 0.5% (w/v) d-glucose and 200 μg/mL thiamphenicol (Sigma-Aldrich). Cell viability was monitored by OD_600nm_ at 0, 2, 4, 10, and 16 h post-treatment. Each condition was tested with two biological replicates and three technical replicates.

#### *In silico* structural modeling and molecular docking

To evaluate the potential interactions between accumulated dipeptides and candidate regulatory proteins, *in silico* structural analyses were performed. The protein sequences of the methyl-accepting chemotaxis protein (MCP, NA23_RS01195), Crp/Fnr family transcriptional regulator (Crp, NA23_RS08105), and D-ribose ABC transporter substrate-binding protein (RbsB, NA23_RS00870) were retrieved from the *F. islandicum* AW-1 genome. Transmembrane domains and signal peptides were predicted using Phobius to identify extracellular or sensory regions. Three-dimensional protein structures were predicted using AlphaFold2 (v2.3.2) via ColabFold.^75^ Potential ligand-binding pockets within the predicted structures were identified using PrankWeb, which utilizes the P2Rank machine learning-based algorithm to rank pockets by their probability scores.^76^ Molecular docking was subsequently performed using GNINA (v1.3.2), an AlphaDock Vina-based tool integrated with a convolutional neural network (CNN) scoring function.^77^ The accumulated dipeptides, Ala-Ile and Gly-Val, were docked into the top-ranked pockets. The docking results were evaluated using three metrics. Vina affinity(kcal/mol) represents the empirical binding energy. The CNN pose score ranges from 0 to 1 and indicates the structural plausibility of the docking configuration. CNN affinity is a deep learning based predicted affinity. The docked poses and protein ligand interactions were visualized using the py3Dmol package.

### Quantification and statistical analysis

The data are reported as the means and standard deviations. Statistical analyses and statistical calculations were performed using Origin 2025b software (OriginLab, USA) and GraphPad 7 (GraphPad Software Inc., USA). *p <* 0.05 was considered statistically significant. Statistical details of each experiment can be found in figure legends, including the test used, number of individual samples (n value), *p* value and definitions.

## Supplemental information

Document S1. Figures S1–S10 and Tables S1–S5

Data S1. Nutrient dependent transcriptomic analysis, related to Figures 2 and 3

Data S2. LC-MS2 proteomic analysis, related to Figure 3D

Data S3. Network analysis, related to Figures 3E and S4

Data S4. Metabolomic analysis, related to Figure 4
