# Supplementary material for: Integrated multiomics reveals starvation-driven keratin degradation and persistence in Fervidobacterium islandicum AW-1
Source: iScience. 2026 Apr 20;29(5):115755. doi: 10.1016/j.isci.2026.115755 (PMC13157175; doi:10.1016/j.isci.2026.115755)
Supplement: Document S1. Figures S1–S10 and Tables S1–S5 [file mmc1.pdf]

iScience, Volume *29*

Supplemental information

Integrated multiomics reveals starvation-driven keratin degradation and persistence in *Fervidobacterium islandicum* AW-1

Jae-Yoon Sung, Ji-Yeon Kim, Hyeon-Su Jin, Je-Hyun Baek, Nicole Enjeh Kim, Hyun Ho Song, Seong-Hun Bong, Yong-Jik Lee, Byoung-Chan Kim, Do Yup Lee, and Dong-Woo Lee

Supplementary information

Table S1. Free amino acid concentration in the culture broth of *F. islandicum* AW-1 with native chicken feathers at 70°C under anaerobic conditions

**Feather (%)**	**Whole feather rachis**		**0.2**		**0.4**		**0.8**		**1.6**		**3.2**	
**Amino acid**	nmol	mol%	nmol	mol %	nmol	mol %	nmol	mol %	nmol	mol %	nmol	mol %
Asp	0.13	5.6	17.38	10.21	18.88	7.73	ND	0.00	10.56	3.35	14.77	5.13
Thr	0.21	4.1	13.71	8.06	20.75	8.49	25.25	9.97	35.98	11.40	22.79	7.91
Ser	0.49	14.1	0.81	0.48	1.76	0.72	4.39	1.73	6.92	2.19	13.38	4.65
Glu	0.38	6.9	ND[a]	0.00	ND	0.00	ND	0.00	ND	0.00	ND	0.00
Gly	0.54	13.7	21.44	12.60	32.62	13.35	47.07	18.58	41.63	13.19	23.72	8.24
Ala	1.05	8.7	64.94	38.17	88.25	36.11	89.74	35.42	115.50	36.60	108.28	37.60
Val	0.36	7.8	23.02	13.53	36.12	14.78	40.41	15.95	55.75	17.66	42.98	14.93
Cys	0.00	8.6	1.24	0.73	2.73	1.12	2.76	1.09	2.98	0.95	4.11	1.43
Met	0.03	0.1	3.36	1.97	3.87	1.58	4.25	1.68	6.98	2.21	10.56	3.67
Ile	0.11	3.2	10.43	6.13	17.49	7.16	18.60	7.34	26.52	8.40	24.89	8.64
Leu	0.14	8.3	ND	0.00	ND	0.00	ND	0.00	ND	0.00	ND	0.00
Tyr	0.12	1.4	5.29	3.11	8.44	3.45	9.81	3.87	ND	0.00	11.51	4.00
Phe	0.07	3.1	ND	0.00	ND	0.00	ND	0.00	ND	0.00	ND	0.00
Lys	0.18	0.6	8.53	5.01	13.49	5.52	11.04	4.36	12.77	4.05	10.98	3.81
His	0.40	0.2	ND	0.00	ND	0.00	ND	0.00	ND	0.00	ND	0.00
Arg	0.24	3.8	ND	0.00	ND	0.00	ND	0.00	ND	0.00	ND	0.00
Pro	1.03	9.8	ND	0.00	ND	0.00	ND	0.00	ND	0.00	ND	0.00
**Total**	5.20	100.0	170.14	100.00	244.40	100.00	253.33	100.00	315.59	100.00	287.96	100.00

Free amino acid analyses in mTF medium with varying feather concentrations after 96 h of anaerobic culture of *F. islandicum* AW-1 at 70°C. The amounts of amino acids in 20 μl of culture supernatant are presented.

[a]ND, not determined.

Table S2. Fatty acid compositions in the cellular membrane of *F. islandicum* AW-1 grown on mTF medium supplemented with 0.5% (w/v) glucose or 0.8% (w/v) native chicken feathers

Fatty acids	Glucose	Feather
$C_{10:0}$ FAME[a]	0.97 ± 0.32	tr[b]
$C_{12:0}$ FAME	-[c]	tr
$C_{14:0}$ FAME	15.64 ± 1.03[d]	2.37 ± 0.06
iso $C_{15:0}$ FAME	-	tr
anteiso $C_{15:0}$ FAME	-	tr
$C_{15:0}$ FAME	-	tr
$C_{16:1}$ *cis*-7 FAME	-	tr
$C_{16:1}$ *cis*-9 FAME	-	2.07 ± 0.08
$C_{16:0}$ FAME	79.06 ± 0.09	44.15 ± 0.21
$C_{17:0}$ FAME	-	1.84 ± 0.05
$C_{18:2}$ *cis*-9, 12 FAME	-	1.54 ± 0.07
$C_{18:1}$ *cis*-9 FAME	1.13 ± 0.19	23.49 ± 0.08
$C_{18:0}$ FAME	3.28 ± 0.93	13.59 ± 0.47
$C_{19:0}$ FAME	-	tr
$C_{20:1}$ *cis*-11 FAME	-	tr
$C_{20:0}$ FAME	-	2.25 ± 0.32
Summed feature 2[e]	-	tr
Summed feature 10[f]	-	2.52 ± 0.08
Summed feature 11[g]	-	tr
Summed feature 12[h]	-	1.11 ± 0.01

[a]FAME, fatty acid methyl ester

[b]tr, trace (<1%)

[c]-, not detected.

[d]Data are expressed as means ± standard deviations (SDs).

[e]Summed feature 2 comprises $C_{12:0}$ 3-0H FAME and/or $C_{13:0}$ dimethyl acetal (DMA).

[f]Summed feature 10 comprises $C_{18:1}$ c11/t9/t6 FAME and/or UN 17.834.

[g]Summed feature 11 comprises iso-$C_{17:0}$ 30H FAME and/or $C_{18:2}$ DMA.

[h]Summed feature 12 comprises UN 18.622 and/or iso-$C_{19:0}$ FAME.

Table S3. A summary of raw reads of RNA-Seq data

Sample ID	Total read bases (bp)	Total reads	Sequencing platform	GC (%)	AT (%)	Q20 (%)	Q30 (%)
FAW1-Fea-1	967,574,302	18,897,624	Illumina HiSeq 2500	43.0	57.0	96.3	92.7
FAW1-Fea-2	2,583,525,864	25,579,464	Illumina NovaSeq 6000	44.7	55.3	98.6	95.5
FAW1-Fea-3	2,495,933,210	24,712,210	Illumina NovaSeq 6000	45.9	54.1	98.7	95.5
FAW1-Pep-1	1,665,824,893	32,705,112	Illumina HiSeq 2500	43.2	56.8	96.5	93.0
FAW1-Pep-2	3,687,524,746	36,510,146	Illumina NovaSeq 6000	44.4	55.6	98.9	96.1
FAW1-Pep-3	2,324,358,450	23,013,450	Illumina NovaSeq 6000	44.1	55.9	98.6	95.3
FAW1-Trp-1	1,100,011,425	21,597,348	Illumina HiSeq 2500	43.8	56.2	96.4	92.9
FAW1-Trp-2	2,226,273,916	22,042,316	Illumina NovaSeq 6000	45.1	54.9	98.6	95.3
FAW1-Trp-3	2,041,061,732	20,208,532	Illumina NovaSeq 6000	44.9	55.1	98.7	95.5
FAW1-Glc-1	1,161,356,801	22,801,919	Illumina HiSeq 2500	43.1	56.9	96.4	92.9
FAW1-Glc-2	2,322,133,218	22,991,418	Illumina NovaSeq 6000	46.5	53.5	98.3	94.6
FAW1-Glc-3	3,665,953,166	36,296,566	Illumina NovaSeq 6000	46.7	53.3	98.5	95.0
FAW1-Fea_12h-1	2,260,657,750	22,382,750	Illumina NovaSeq 6000	44.6	55.4	97.7	93.8
FAW1-Fea_12h-2	3,143,896,892	31,127,692	Illumina NovaSeq 6000	50.1	49.9	97.6	93.8
FAW1-Fea_12h-3	3,161,199,808	31,299,008	Illumina NovaSeq 6000	45.6	54.4	97.7	93.8

Table S4. Primers used for qRT-PCR analysis

Gene name	Sequence (5'-3')	Tm (°C)
RS02690 (*che*A)	AGAGGGTGAACCACAAAAGG	59.0
	CTTCGTGCAATCACAAGCTC	59.6
RS01010 (*che*C)	ATCTGTCCCGCAAGTAAAGG	59.2
	TTTGGGTCGAAGATCAGCAG	61.3
RS01015 (*che*D)	CACGGGTGTTAATCTTGTGG	58.9
	ACCGCTTCTACATTCCTTGC	59.3
RS06960 (*che*R)	ACGGCACAATCTTCTCCAAG	60.3
	CGAATAATAGTCCGCCAACG	60.5
RS06950 (*che*Y)	CGAAAGATGGGATGGAACAG	60.5
	ACTCCACAGCCTTTTCAACG	60.3
RS01195 (*mcp*)	AAACTCGCAACTGCCTTGAG	60.6
	TCTTCGATTGTCGCACTGAC	60.0
RS01020 (*fli*A)	TGCCAAAAACCAGCGACTAC	61.2
	CTTCTTCGTCCGAACCAAAC	59.7
RS09605 (*fli*E)	GGTGGGGTTAATCCGTTAAG	58.3
	CGGTCAACTTTTCCACGTTC	60.5
RS02780 (*fli*I)	ATCGGTGAACCTCCAACAAC	59.8
	ATCGGCTTCGACAAGAACTG	60.4
RS05380 (*flg*A)	TCGTTGCATACGTTCCTGTC	59.7
	GAGCCCTTTGTTGTGCTTTC	59.9
RS09600 (*flg*C)	GGCGCAAAGGTTCAGAATAG	59.8
	ACCAGAGTTTTCACGCTTGC	60.4
RS06620 (*flg*G)	TTTGGCTATCAGTGGTGACG	59.7
	AGAGATACCGCGTTTTGTGG	60.1
RS09955 (*flg*M)	CGGAGGTCAAAGGAAAAACC	60.8
	ATACTCGGCCACTTTCCTTC	58.3
RS06825 (*dgc*)	TCTTTCTTGCCCTCAGGTTC	59.4
	CTGCTGTATGCACCGGTTAG	59.4
RS00930 (*obg*E)	CGCAGAGAATGGAGAAAACG	60.9
	TATTTCCCAGGCTCGTCAAG	60.2
RS06865 (*rel*)	TCCGAGTTGGAGGATTTGAG	60.2
	TGCTCTTGGAACGATGTCTG	60.0
RS05650 (*ndk*)	AGGGAAGCCGTTTTACCAAG	60.5
	TGCTCCGACTATGTGCCTTAC	60.3
RS06785 (*dgc*)	AGATACGGAGGAGACGAGTTTG	59.8
	TCGACTAAGGTTTGCCCTTG	60.2
RS02930 (*eal*)	TCACGAGCAAAGTCGTCAAG	60.2
	TCTTCAGCTCGCCATTGTAG	59.2
RS04380 (*hd-gyp*)	TCACCTTTCTCATCGGCTTC	60.3
	TCAACGTATTCACCGGTCTC	58.6
RS05240 (*pfs*)	GCGTTCGCAGTTGATATGG	60.2
	TAATGGCAGAGGGCAAACTC	60.2
RS06465 (*psm*)	AATCCGGAGGACTTGAAAGC	60.6
	CCATTGCACTTGACCATGAC	60.0

RS04350 (*fab*Z)	CGAGGCAAGGTTCAAGAAAG	60.0
	TCACCGACCTTAGCTTTTCC	59.3
RS02100 (*hfq*)	GGGGATTGTTAGGTCGTTTG	59.3
	GTTCCGATTCTTGCTCTTCG	60.0
RS00870 (*rbs*B)	TCTCCGTCTTTCTTCCTTGC	60.0
	TCGCTGCAGATAGTGCAAAG	60.3
RS10010 (*lux*R)	CGAAGAGATCCTCGAAATGG	59.8
	TCTCCGTCTTTCTTCCTTGC	59.5
RS08105 (*crp*/*fnr*R)	CAGCGGGAACGAAAATCTAC	59.7
	GATGGCAGGTTGAAAAGAGC	59.8
RS00485 (*rpo*D)	TGTGGGTGGTGAGAAGATTG	59.5
	AGCTTCTTCATCACCCTTCG	59.4

Flagellar biosynthesis (9 genes), chemotaxis (6 genes), ppGpp and C-di-GMP signaling (8 genes), autoinducer production (3 genes), and transcriptional regulation (4 genes)-related genes were analyzed for *F. islandicum* AW-1 after 3, 4, 5, 6, 8, and 12 hours of anaerobic culture in mTF+0.8% fea medium at 70°C. The Tm values were calculated using Primer3Plus.

Table S5. Structure-based docking summary for dipeptide binding to candidate sensing/regulatory proteins in *F. islandicum* AW-1

Target	Ligand	Vina affinity (kcal/mol)	CNN pose score	CNN affinity	Product
MCP sensory domain	Ala-Ile	-5.61	0.8995	4.416	methyl-accepting chemotaxis protein (MCP)
MCP sensory domain	Gly-Val	-5.18	0.8468	4.022	methyl-accepting chemotaxis protein (MCP)
Crp/Fnr family regulator	Ala-Ile	-6.01	0.6228	4.642	Crp/Fnr family transcriptional regulator (Crp)
Crp/Fnr family regulator	Gly-Val	-5.59	0.7353	4.574	Crp/Fnr family transcriptional regulator (Crp)
RbsB (substrate-binding protein)	Ala-Ile	-4.92	0.4447	3.516	D-ribose ABC transporter substrate-binding protein (RbsB)
RbsB (substrate-binding protein)	Gly-Val	-4.44	0.9144	3.896	D-ribose ABC transporter substrate-binding protein (RbsB)

Vina affinity indicates docking score. CNN pose score (0 –1) reflects the likelihood of a structurally plausible low-RMSD pose as reported by GNINA. CNN affinity indicates the GNINA CNN-predicted affinity metric.

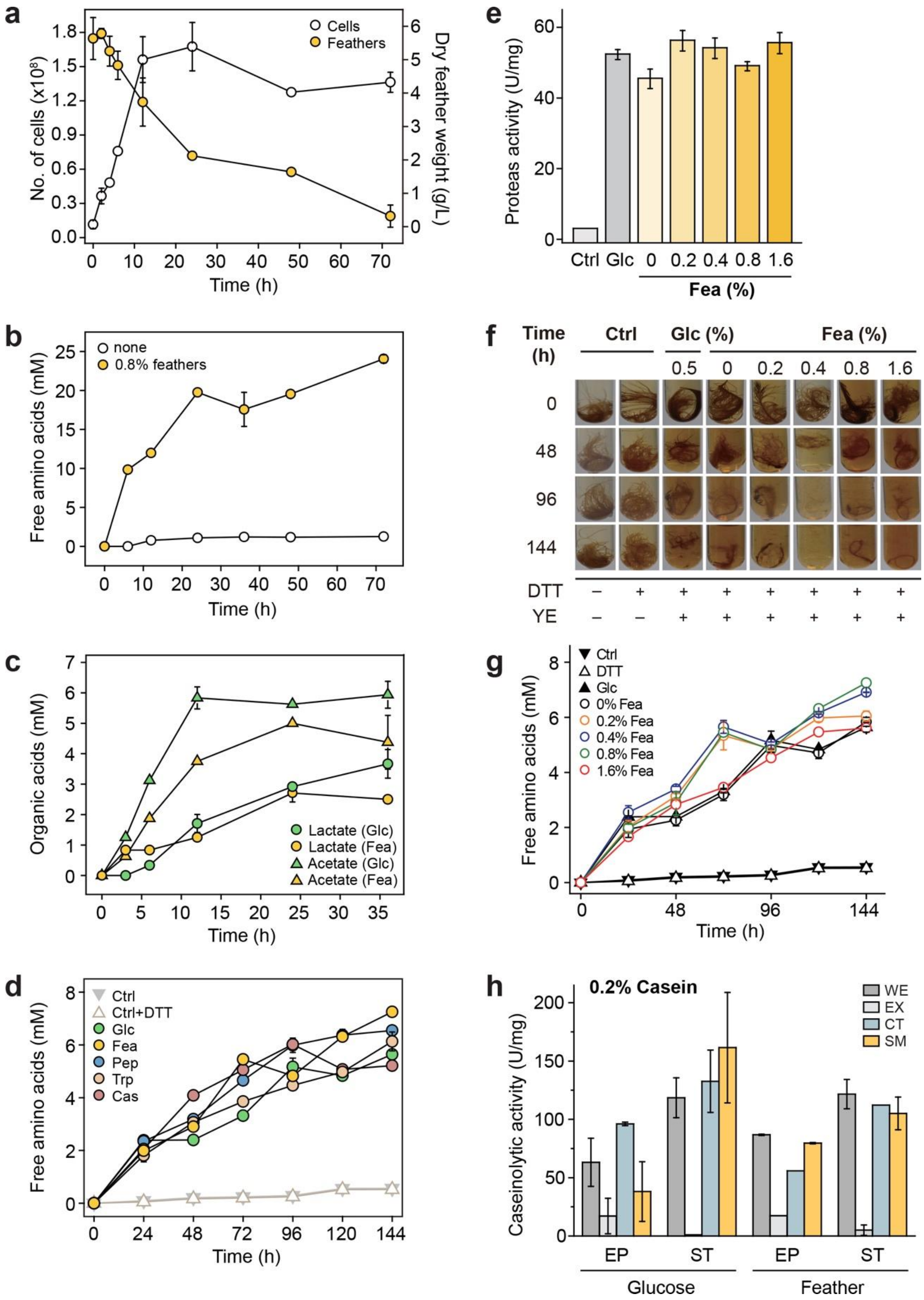

Figure S1. Nutrient- and redox-dependent keratin degradation by *F. islandicum* AW-1. (a) Time-course of cell growth and feather degradation in mTF medium supplemented with 0.8% native chicken

feathers at 70°C and pH 7.0. Feather biomass was quantified by dry weight. (**b**) Accumulation of free amino acids in cultures with 0.8% feathers (yellow) or no supplement (open circles). (**c**) Organic acid production during growth on 0.8% feathers (yellow) vs. 0.5% glucose (green). (**d**) Free amino acid release from cells grown on various nutrient conditions (Glc, Fea, Pep, Trp, Cas), with or without 10 mM DTT. (**e**) Total protease activity in crude extracts from cells grown on Glc or increasing concentrations of Fea (0.2–1.6%, w/v). Data represent means ± SD (n=3). (**f**) Time-course of feather degradation using crude extracts from glucose- or feather-grown cells, with or without DTT supplementation. Reactions were carried out at 75 °C and pH 7.0 for 144 h. (**g**) Keratinolytic activity at different feather concentrations, measured by the release of free amino acids from native feathers over 144 h at 75°C. (**h**) Caseinolytic activity of subcellular fractions (WE, EX, CT, SM) from exponential (EP) and stationary (ST) phase cells grown on Glc or Fea. Caseinolytic activity was conducted using 0.2% casein at 90°C and pH 7.0 for 20 min.

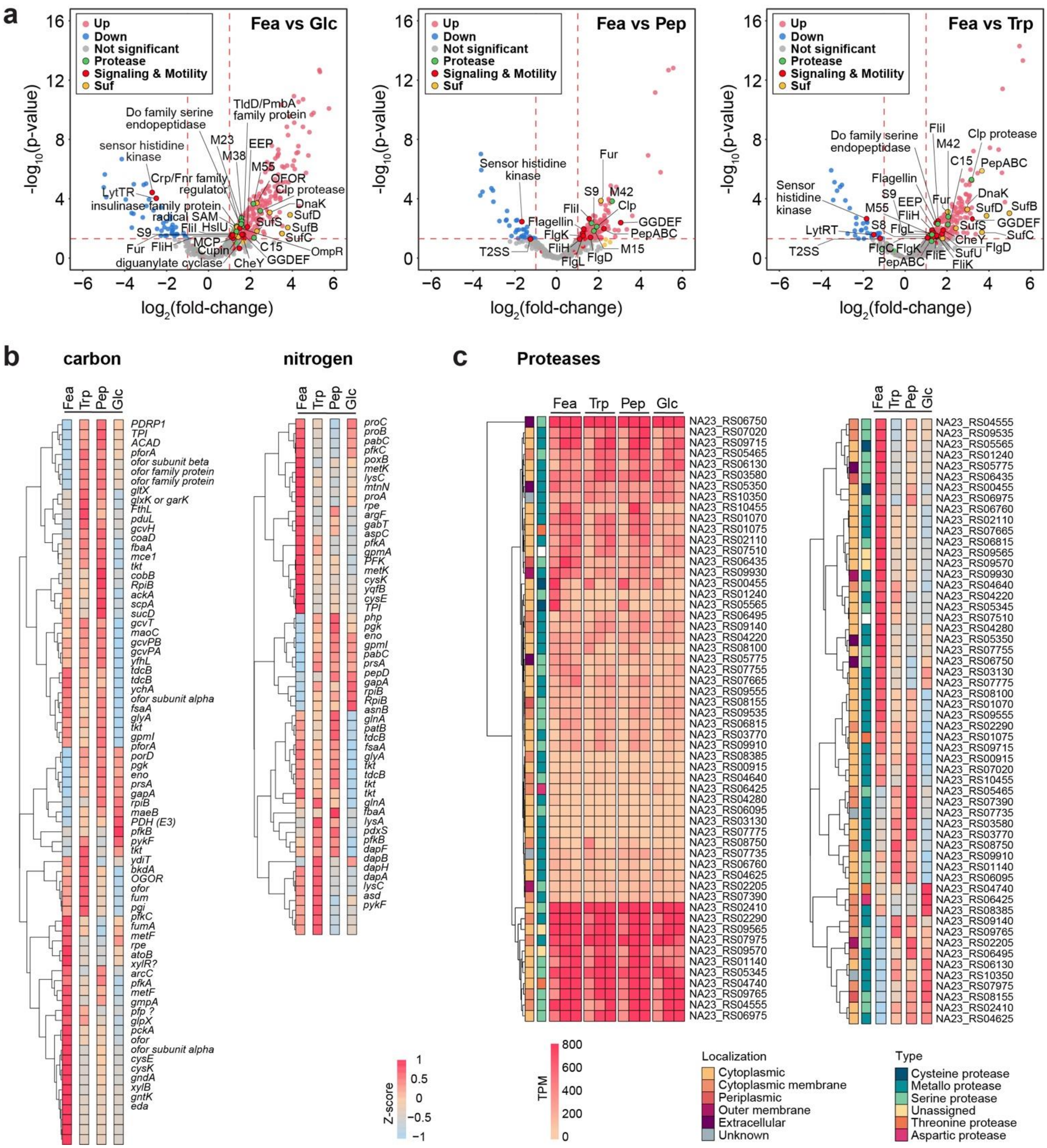

Figure S2. Nutrient-specific transcriptomic responses and protease gene expression in *F. islandicum* AW-1. (**a**) Volcano plots comparing DEGs from cells grown on feathers (Fea), peptone (Pep), or tryptone (Trp) relative to glucose (Glc). All samples are biologically replicated in triplicate. Red dots indicate significantly upregulated genes (≥ 2.0 fold, $p \leq 0.05$). Blue dots indicate significantly downregulated genes (≤ -2.0 fold, $p \leq 0.05$). Grey dots represent non-significant genes ($p > 0.05$).

Functionally relevant genes related to proteases, signaling & motility, and sulfur metabolism (Suf) are highlighted. (**b**) Heatmaps of DEGs involved in nitrogen and carbon metabolism. Genes involved in nitrogen metabolism (left) and carbon metabolism (right) are clustered based on their expression levels. Red indicates high expression, while blue represents low expression (Z-score normalized). (**c**) Heatmaps of 57 protease-encoding genes under different nutrient conditions. The heatmap on the left shows the expression of protease genes categorized by cellular localization (cytoplasmic, periplasmic, outer membrane, extracellular). The heatmap on the right classifies proteases by enzyme type, including cysteine, metalloprotease, serine, and threonine proteases. Expression levels are shown in transcripts per million (TPM).

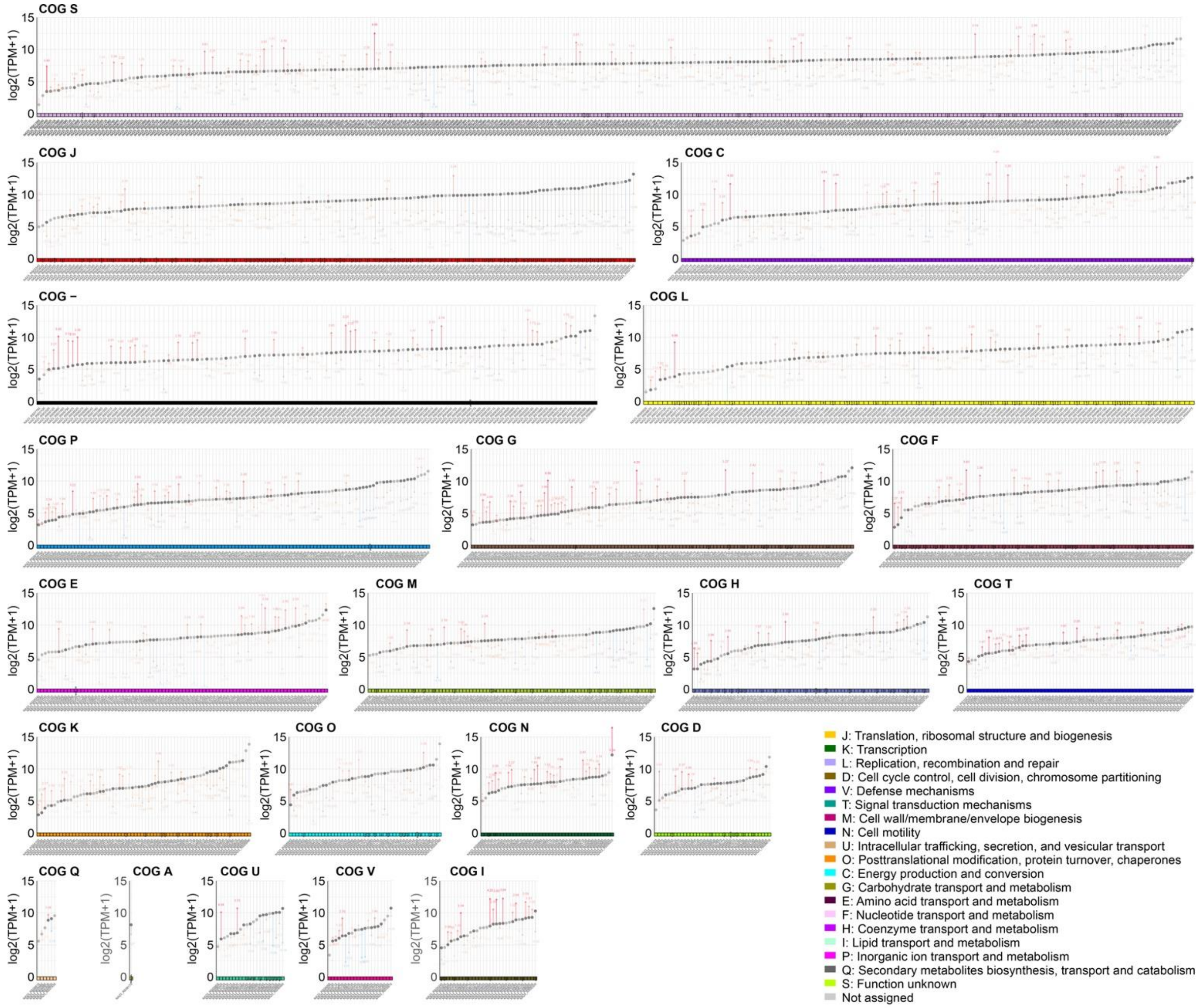

Figure S3. Functional category-specific transcriptomic changes during late-stage keratin degradation. COG-based expression profiles comparing Fea-grown cells at 12 h vs. 8 h. Genes are grouped by COG category, and their expression is shown as $\log_2$(TPM + 1). Vertical arrows represent relative transcript changes at 12 h compared to 8 h: red for upregulated genes, blue for downregulated. Significant induction is observed in stress response (O), translation (J), membrane biogenesis (M), and energy metabolism (E, G, P), indicating a transition to a stress-adaptive state during prolonged feather degradation.

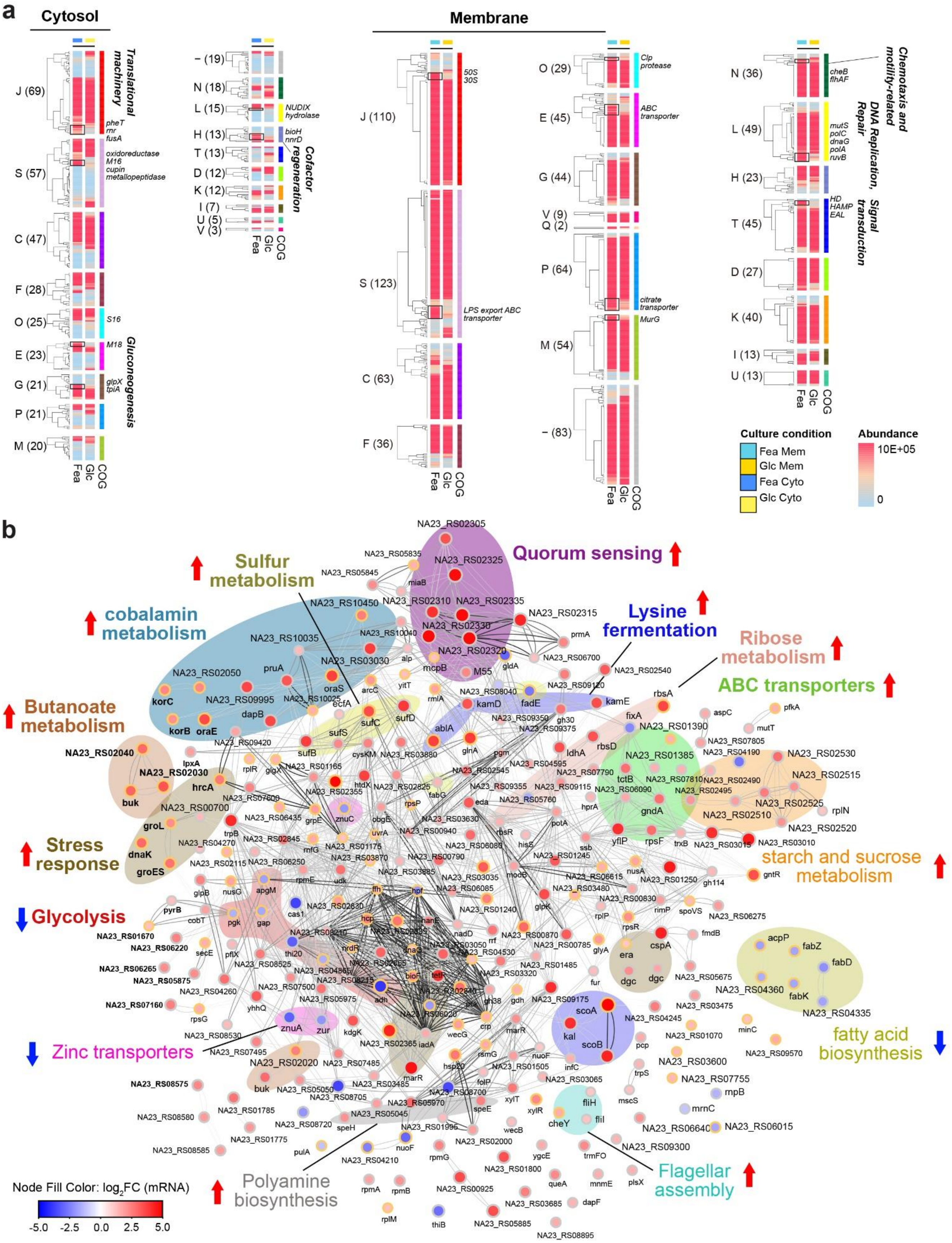
a
Cytosol
Membrane
Translational machinery
Cofactor regeneration
Gluconeogenesis
Chemotaxis and motility-related
DNA Replication, Repair
Signal transduction
Culture condition
Fea Mem
Glc Mem
Fea Cyto
Glc Cyto
Abundance
10E+05
0
b
Quorum sensing
Sulfur metabolism
cobalamin metabolism
Lysine fermentation
Ribose metabolism
ABC transporters
Butanoate metabolism
Stress response
starch and sucrose metabolism
Glycolysis
Zinc transporters
fatty acid biosynthesis
Polyamine biosynthesis
Flagellar assembly
Node Fill Color: log2FC (mRNA)
-5.0 -2.5 0.0 2.5 5.0

Figure S4. Proteomic comparison and regulatory protein-protein interaction (PPI) networks underlying starvation-induced keratinolysis in *F. islandicum* AW-1. (**a**) Heatmaps of Z-score normarlized abundances of DEPs in cytosolic and membrane-associated fractions from Fea-grown and Glc cells, grouped by COG functional categories. Functional groups include proteolysis, transport, chemotaxis/motility, stress responses, and sulfur metabolism. (**b**) Global PPI network derived from transcriptomic DEGs ($log_2FC > 1.0$, $p < 0.05$, STRING combined score > 400; n = 291) comparing Fea- versus Glc-grown cells. Nodes are colored by transcript expression change (red: upregulated, blue: downregulated), with black-bordered nodes representing proteins also identified in the proteomic dataset. KEGG pathway annotation highlights functional modules involved in proteolysis, motility, sulfur metabolism, ABC transporters, chemotaxis, nutrient scavenging, and stress adaptation.

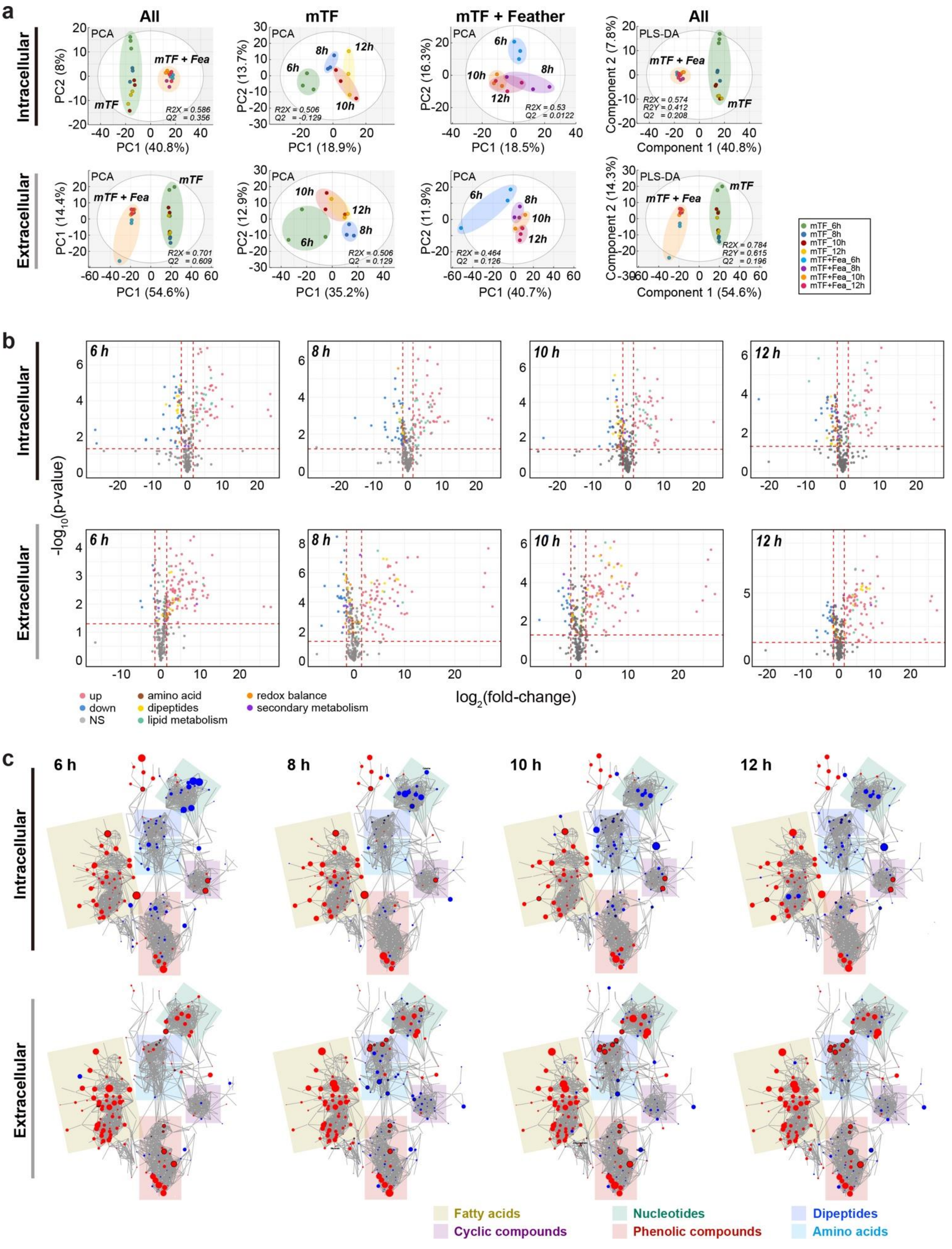
a
b
c
All
mTF
mTF + Feather
Intracellular
Extracellular
PCA
PLS-DA
mTF + Fea
PC1 (40.8%)
PC2 (8%)
R2X = 0.586
Q2 = 0.356
PC1 (18.9%)
PC2 (13.7%)
R2X = 0.506
Q2 = -0.129
PC1 (18.5%)
PC2 (16.3%)
R2X = 0.53
Q2 = 0.0122
Component 1 (40.8%)
Component 2 (7.8%)
R2X = 0.574
R2Y = 0.412
Q2 = 0.208
PC1 (54.6%)
PC1 (14.4%)
R2X = 0.701
Q2 = 0.609
PC1 (35.2%)
PC2 (12.9%)
R2X = 0.506
Q2 = 0.129
PC1 (40.7%)
PC2 (11.9%)
R2X = 0.464
Q2 = 0.126
Component 1 (54.6%)
Component 2 (14.3%)
R2X = 0.784
R2Y = 0.615
Q2 = 0.196
mTF_6h
mTF_8h
mTF_10h
mTF_12h
mTF+Fea_6h
mTF+Fea_8h
mTF+Fea_10h
mTF+Fea_12h
6 h
8 h
10 h
12 h
-log10(p-value)
log2(fold-change)
up
down
NS
amino acid
dipeptides
lipid metabolism
redox balance
secondary metabolism
Fatty acids
Cyclic compounds
Nucleotides
Phenolic compounds
Dipeptides
Amino acids

Figure S5. Temporal metabolic reprogramming during feather degradation in *F. islandicum* AW-1. (**a**) Multivariatde analysis (PCA and PLS-DA) of intracellular (top) and extracellular (bottom) metabolite profiles from cells cultured in mTF medium ± feathers at 6, 8, 10, and 12 h. Clustering and separation of feather-supplemented cultures indicate distinct metabolic reorganization under nutrient limitation. (**b**) Volcano plots of significantly altered metabolites at each time point (intracellular, top; extracellular, bottom). Metabolites with FC ≥ 2 and $p < 0.05$ were classified as upregulated (red) or downregulated (blue), while non-significant features are shown in grey. **(c)** Metabolite networks of intracellular (top) and extracellular (bottom) fractions clustered by chemical class with colored by fold-change direction (red: upregulated; blue: downregulated; gray: unchanged). Shaded areas highlight major compound classes such as fatty acids, cyclic compounds, nucleotides, phenolics, dipeptides, and amino acids. These temporal profiles indicate early depletion of amino acids/dipeptides followed by accumulation of fatty acids and secondary metabolites, consistent with energy conservation and redox balancing under nutrient limitation.

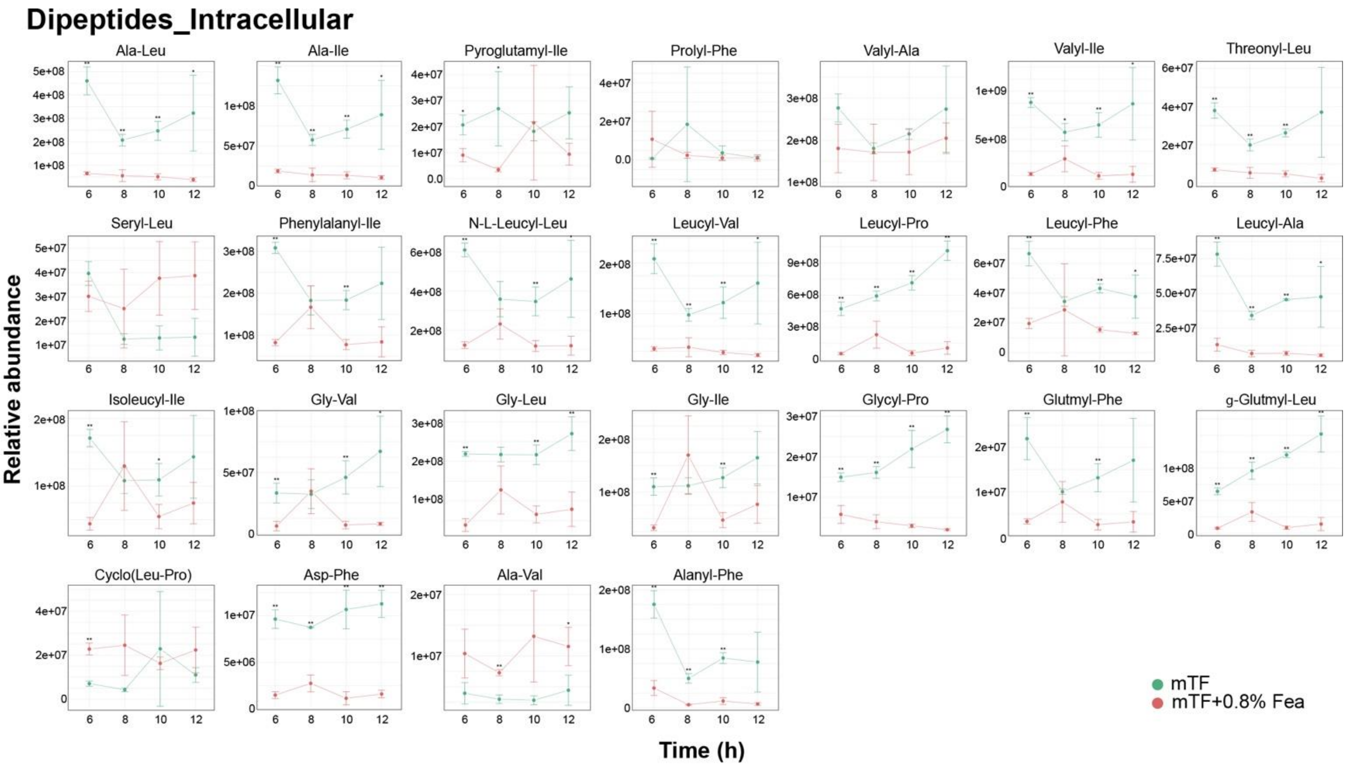
Dipeptides_Intracellular
Ala-Leu
Ala-Ile
Pyroglutamyl-Ile
Prolyl-Phe
Valyl-Ala
Valyl-Ile
Threonyl-Leu
Seryl-Leu
Phenylalanyl-Ile
N-L-Leucyl-Leu
Leucyl-Val
Leucyl-Pro
Leucyl-Phe
Leucyl-Ala
Isoleucyl-Ile
Gly-Val
Gly-Leu
Gly-Ile
Glycyl-Pro
Glutmyl-Phe
g-Glutmyl-Leu
Cyclo(Leu-Pro)
Asp-Phe
Ala-Val
Alanyl-Phe
Relative abundance
Time (h)
mTF
mTF+0.8% Fea

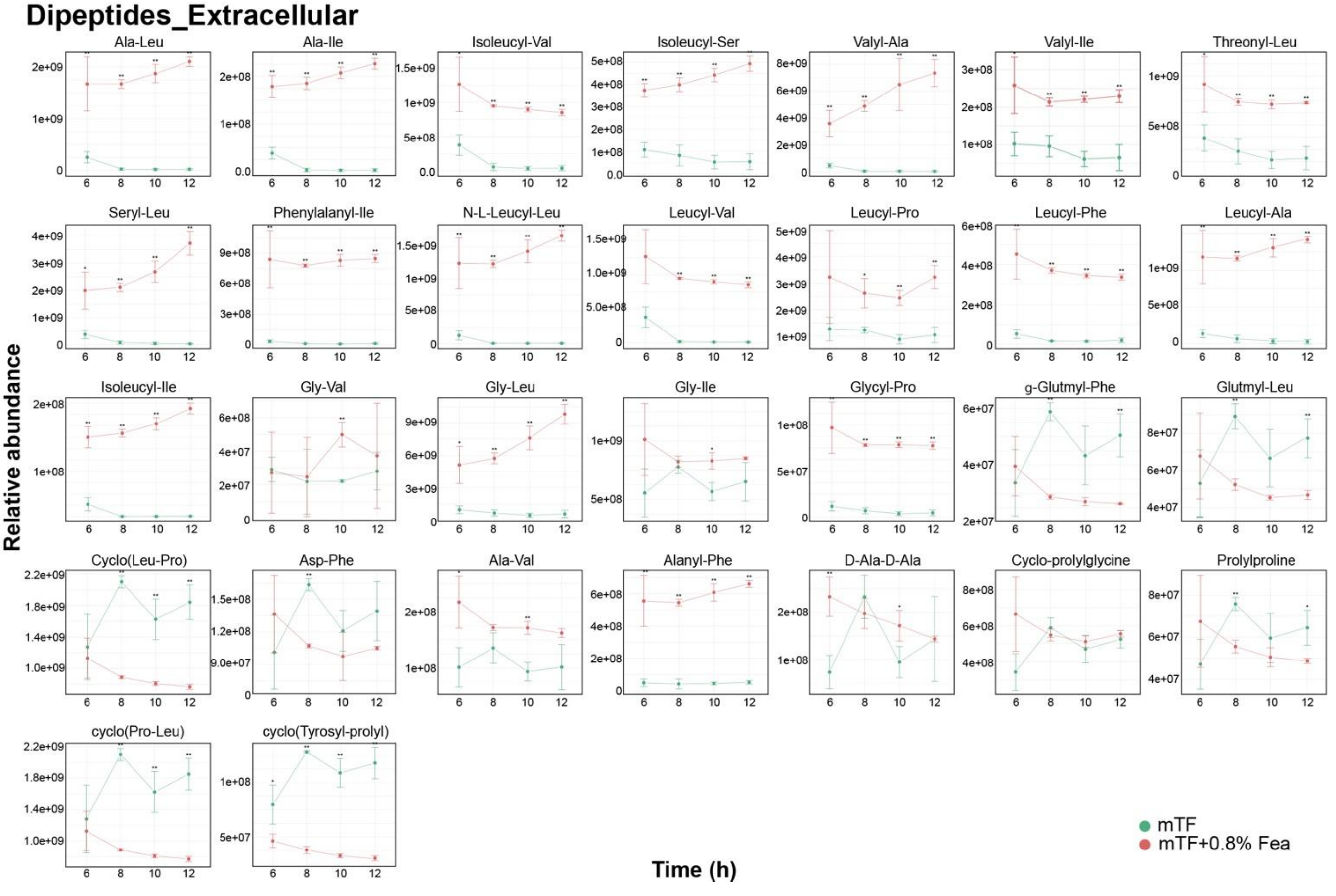
Dipeptides_Extracellular
Ala-Leu
Ala-Ile
Isoleucyl-Val
Isoleucyl-Ser
Valyl-Ala
Valyl-Ile
Threonyl-Leu
Seryl-Leu
Phenylalanyl-Ile
N-L-Leucyl-Leu
Leucyl-Val
Leucyl-Pro
Leucyl-Phe
Leucyl-Ala
Isoleucyl-Ile
Gly-Val
Gly-Leu
Gly-Ile
Glycyl-Pro
g-Glutmyl-Phe
Glutmyl-Leu
Cyclo(Leu-Pro)
Asp-Phe
Ala-Val
Alanyl-Phe
D-Ala-D-Ala
Cyclo-prolylglycine
Prolylproline
cyclo(Pro-Leu)
cyclo(Tyrosyl-prolyl)
Relative abundance
Time (h)
mTF
mTF+0.8% Fea

Figure S6. Temporal metabolomic profiling of dipeptides during feather degradation by *F. islandicum* AW-1. Time-resolved intracellular and extracellular abundances of dipeptides measured at 6, 8, 10, and 12 h during growth in mTF medium supplemented with or without 0.8% (w/v) feathers. Each panel displays relative abundances (log scale) of individual metabolites, grouped by compound class and compartment (intra-/extracellular). Data are shown as mean ± SD (n = 3). Metabolites were selected based on significant temporal changes or nutrient-dependent differences (adjusted $p < 0.05$).

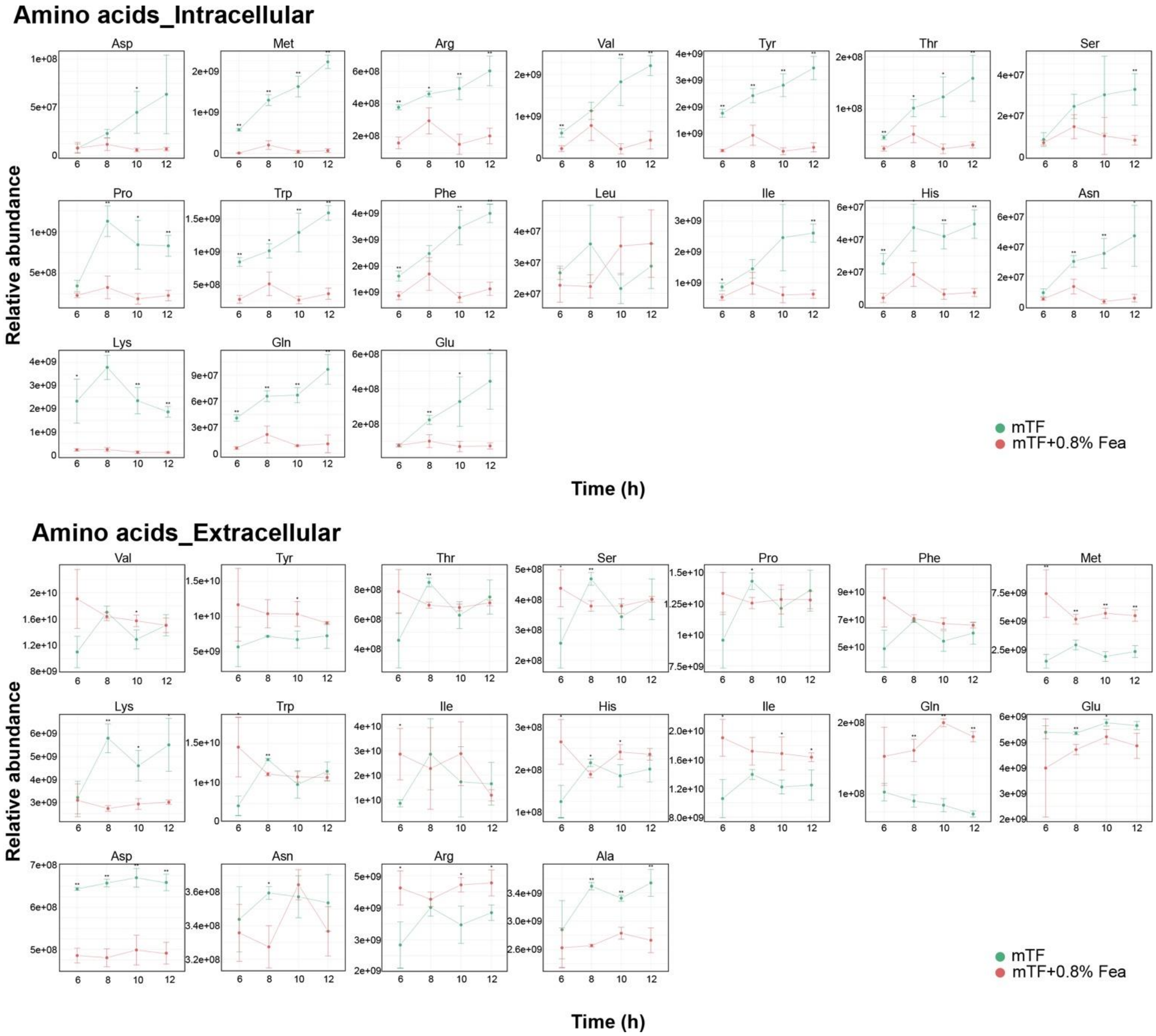

Figure S7. Temporal metabolomic profiling of amino acids during feather degradation by *F. islandicum* AW-1. Time-resolved intracellular and extracellular abundances of amino acids measured at 6, 8, 10, and 12 h during growth in mTF medium supplemented with or without 0.8% (w/v) feathers. Each panel displays relative abundances (log scale) of individual metabolites, grouped by compound class and compartment (intra-/extracellular). Data are shown as mean ± SD (n = 3). Metabolites were selected based on significant temporal changes or nutrient-dependent differences (adjusted $p < 0.05$).

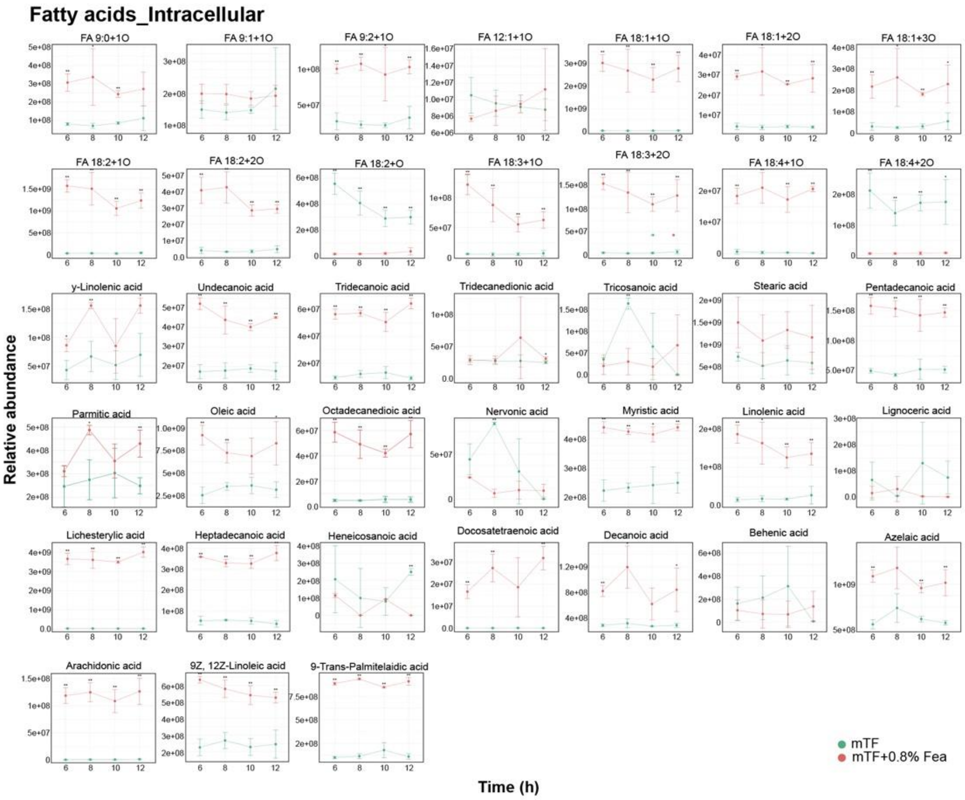
Fatty acids_Intracellular
FA 9:0+1O
FA 9:1+1O
FA 9:2+1O
FA 12:1+1O
FA 18:1+1O
FA 18:1+2O
FA 18:1+3O
FA 18:2+1O
FA 18:2+2O
FA 18:2+O
FA 18:3+1O
FA 18:3+2O
FA 18:4+1O
FA 18:4+2O
y-Linolenic acid
Undecanoic acid
Tridecanoic acid
Tridecanedionic acid
Tricosanoic acid
Stearic acid
Pentadecanoic acid
Parmitic acid
Oleic acid
Octadecanedioic acid
Nervonic acid
Myristic acid
Linolenic acid
Lignoceric acid
Lichesterylic acid
Heptadecanoic acid
Heneicosanoic acid
Docosatetraenoic acid
Decanoic acid
Behenic acid
Azelaic acid
Arachidonic acid
9Z, 12Z-Linoleic acid
9-Trans-Palmitelaidic acid
Relative abundance
Time (h)
mTF
mTF+0.8% Fea

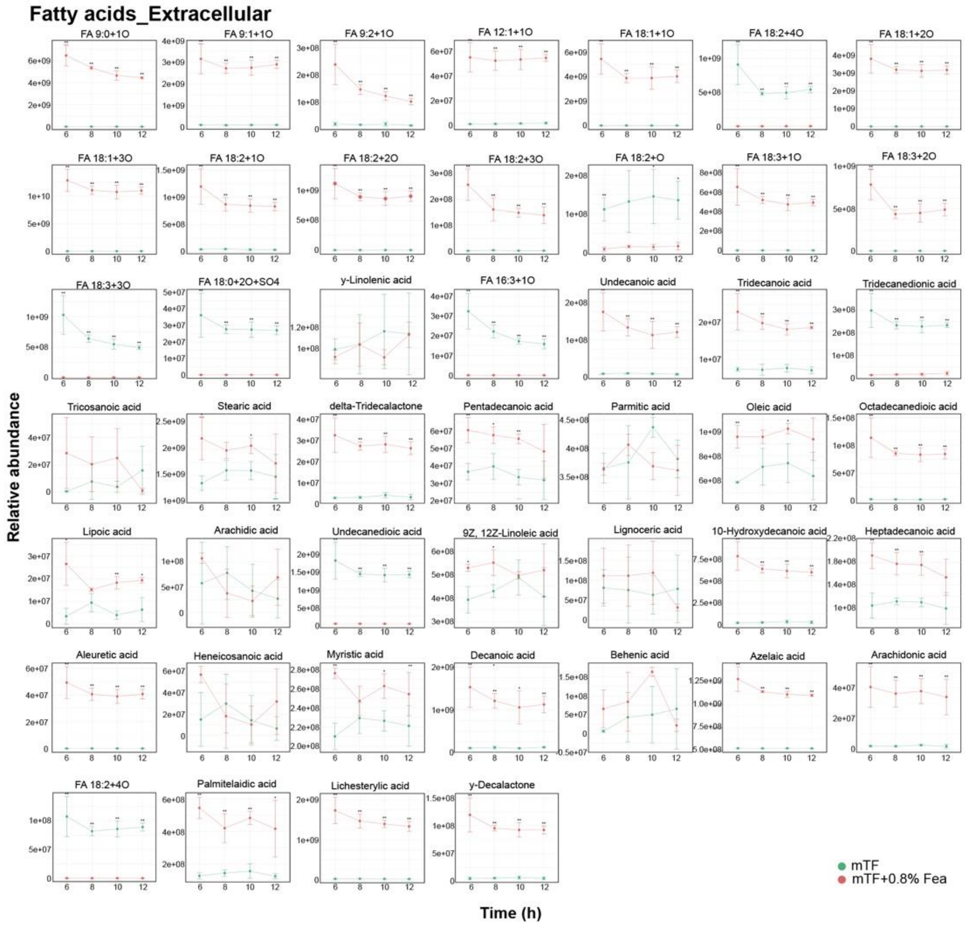
Fatty acids_Extracellular
FA 9:0+1O
FA 9:1+1O
FA 9:2+1O
FA 12:1+1O
FA 18:1+1O
FA 18:2+4O
FA 18:1+2O
FA 18:1+3O
FA 18:2+1O
FA 18:2+2O
FA 18:2+3O
FA 18:2+O
FA 18:3+1O
FA 18:3+2O
FA 18:3+3O
FA 18:0+2O+SO4
y-Linolenic acid
FA 16:3+1O
Undecanoic acid
Tridecanoic acid
Tridecanedionic acid
Tricosanoic acid
Stearic acid
delta-Tridecalactone
Pentadecanoic acid
Parmitic acid
Oleic acid
Octadecanedioic acid
Lipoic acid
Arachidic acid
Undecanedioic acid
9Z, 12Z-Linoleic acid
Lignoceric acid
10-Hydroxydecanoic acid
Heptadecanoic acid
Aleuretic acid
Heneicosanoic acid
Myristic acid
Decanoic acid
Behenic acid
Azelaic acid
Arachidonic acid
FA 18:2+4O
Palmitelaidic acid
Lichesterylic acid
y-Decalactone
Relative abundance
Time (h)
mTF
mTF+0.8% Fea

Figure S8. Temporal metabolomic profiling of fatty acids during feather degradation by *F. islandicum* AW-1. Time-resolved intracellular and extracellular abundances of fatty acids measured at 6, 8, 10, and 12 h during growth in mTF medium supplemented with or without 0.8% (w/v) feathers. Each panel displays relative abundances (log scale) of individual metabolites, grouped by compound class and compartment (intra-/extracellular). Data are shown as mean ± SD (n = 3). Metabolites were selected based on significant temporal changes or nutrient-dependent differences (adjusted $p < 0.05$).

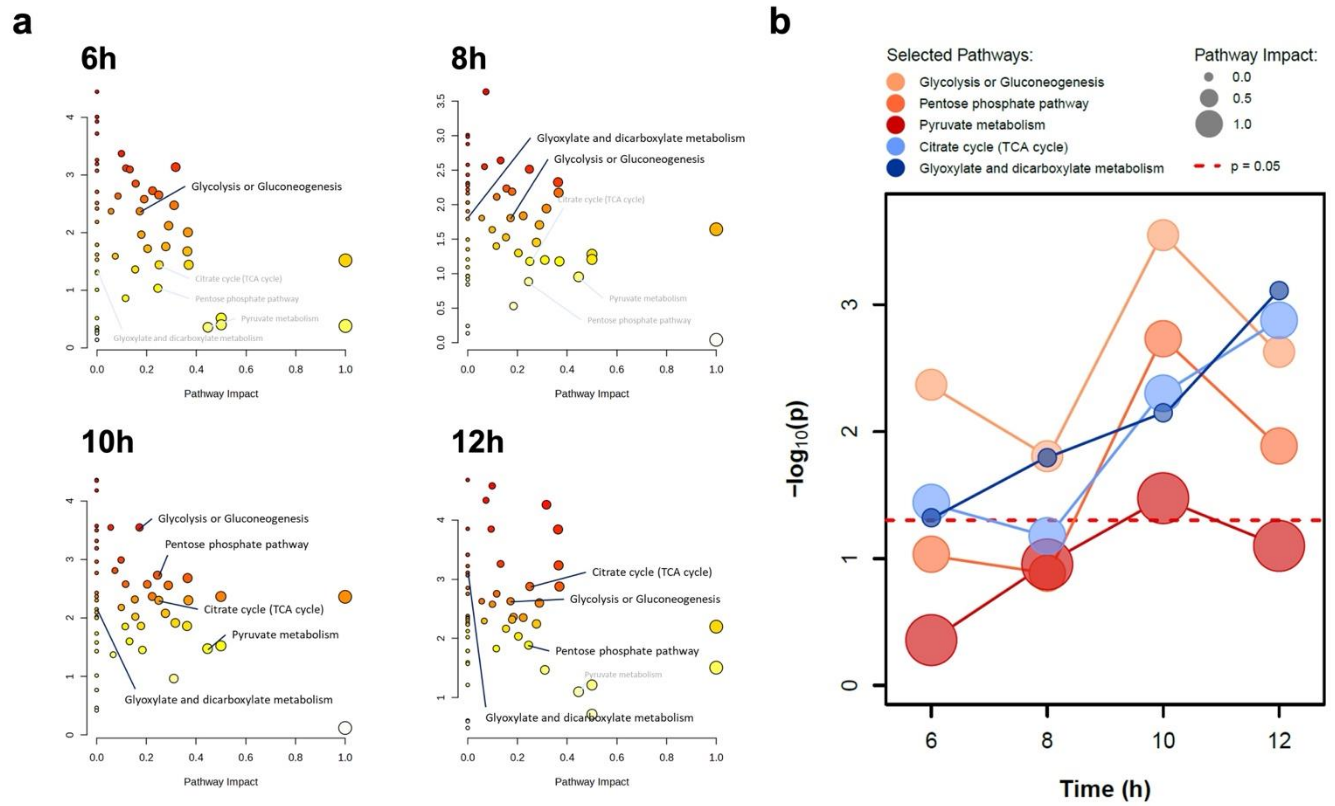

Figure S9. Pathway enrichment analysis of timely-resolved metabolic profiles in *F. islandicum* AW-1. (a) Metabolic pathway enrichment analysis (6, 8, 10, and 12 h). X and Y axis present topological impact score and statistical significance (-log10(P-value), respectively. **(b)** Time-resolved line plots of central carbon metabolism (glycolysis/gluconeogenesis, pentose phosphate pathway, pyruvate metabolism, TCA cycle, and glyoxylate-dicarboxylate metabolism). Circle sizes indicate pathway impact.

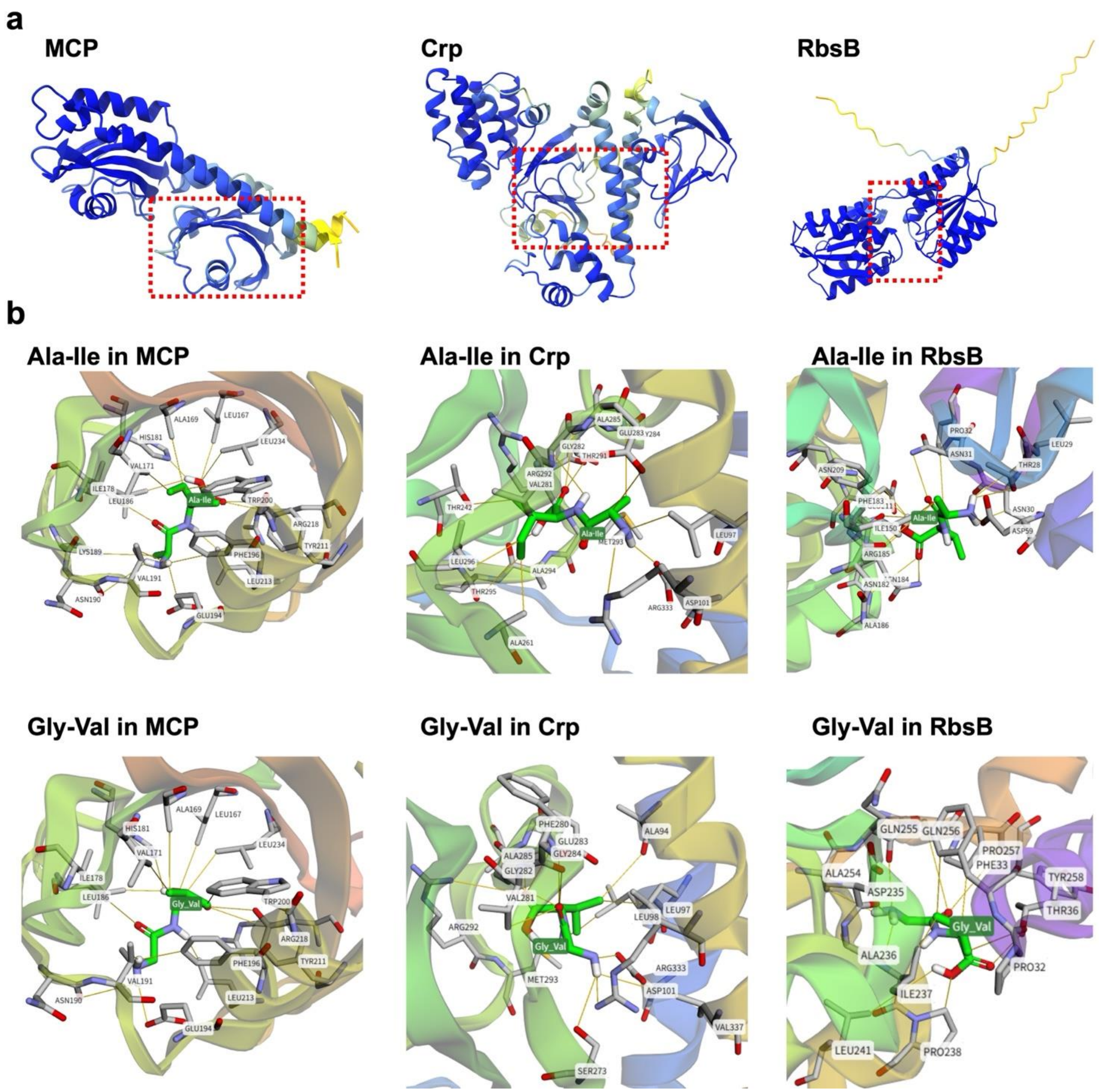

Figure S10. Structure-based docking analysis of accumulated dipeptides with candidate sensing and regulatory proteins. (**a**) AlphaFold2-predicted structures of candidate proteins from *F. islandicum* AW-1, including a methyl-accepting chemotaxis protein (MCP; NA23_RS01195), a Crp/Fnr family transcriptional regulator (NA23_RS08105), and the D-ribose ABC transporter substrate-binding protein RbsB (NA23_RS00870). (**b**) Representative docking poses of Ala–Ile and Gly–Val within predicted ligand-binding pockets, visualized using py3Dmol.
